# Supplementary material for: Non-volatile magnon transport in a single domain multiferroic
Source: Nat Commun. 2024 Jul 16;15:5966. doi: 10.1038/s41467-024-50180-9 (PMC11252442; doi:10.1038/s41467-024-50180-9)
Supplement: Supplementary file 1 — Supplementary Information [file 41467_2024_50180_MOESM1_ESM.pdf]

Supplementary Information
Non-volatile magnon transport in a single domain multiferroic

Sajid Husain[1,†,*], Isaac Harris[1,2,†,], Peter Meisenheimer[3], Sukriti Mantri[4], Xinyan Li[5] Maya Ramesh[6], Piush Behera[1,3], Hossein Taghinejad[2,7], Jaegyu Kim[3], Pravin Kavle[1,3], Shiyu Zhou[8], Tae Yeon Kim[3], Hongrui Zhang[1,3], Paul Stevenson[9], James G. Analytis[2], Darrell Schlom[6], Sayeef Salahuddin[3,10], Jorge Íñiguez-González[11,12], Bin Xu[13], Lane W. Martin[1,3,5,14,15], Lucas Caretta[16], Yimo Han[5], Laurent Bellaiche[4,17], Zhi Yao [18,*], Ramamoorthy Ramesh [1,2,3,5,14,15*]

[1]*Materials Science Division, Lawrence Berkeley National Laboratory, Berkeley, CA, 94720, USA*
[2]*Department of Physics, University of California, Berkeley, CA, 94720, USA*
[3]*Department of Materials Science and Engineering, University of California, Berkeley, CA, 94720, USA*
[4]*Physics Department and Institute for Nanoscience and Engineering, University of Arkansas, Fayetteville, Arkansas 72701, USA*
[5]*Materials Science and NanoEngineering, Rice University, Houston, Texas, 77005, USA.*
[6]*Department of Materials Science and Engineering, Cornell University, Ithaca, NY, 14850, USA*
[7]*Heising-Simons Junior Fellow, Kavli Energy NanoScience Institute (ENSI), University of California, Berkeley, CA, 94720, USA*
[8]*Department of Physics, Brown University, Providence, RI, 02906, USA*
[9]*Department of Physics, Northeastern University, Boston, MA, 02115, USA*
[10]*Department of Electrical Engineering and Computer Sciences, University of California, Berkeley, CA 94720, USA*
[11]*Department of Materials Research and Technology, Luxembourg Institute of Science and Technology, 5 Avenue des Hauts-Fourneaux, L-4362 Esch/Alzette, Luxembourg*
[12]*Department of Physics and Materials Science, University of Luxembourg, 41 Rue du Brill, L-4422 Belvaux, Luxembourg*
[13]*Institute of Theoretical and Applied Physics, Jiangsu Key Laboratory of Thin Films, School of Physical Science and Technology, Soochow University, Suzhou 215006, China*
[14]*Departments of Chemistry, and Physics and Astronomy, Rice University, Houston, TX, 77005, USA.*
[15]*Rice Advanced Materials Institute, Rice University, Houston, TX, 77005, USA.*
[16]*School of Engineering, Brown University, Providence, RI, 77005, USA*
[17]Department of Materials Science and Engineering, Tel Aviv University, Ramat Aviv, Tel Aviv 6997801, Israel.
[18]Applied Mathematics and Computational Research Division, Lawrence Berkeley National Laboratory, Berkeley, CA, 94720, USA
*rramesh@berkeley.edu
*jackie-zhiyao@lbl.gov
*shusain@lbl.gov
† These authors contributed equally

CONTENTS

SUPPLEMENTARY NOTE 1
X-RAY DIFFRACTION 3

SUPPLEMENTARY NOTE 2
CROSS-SECTIONAL MICROSCOPY IMAGING AND POLARIZATION MAPPING 6

SUPPLEMENTARY NOTE 3
FERROELECTRIC DOMAIN CHARACTERISTICS 8

SUPPLEMENTARY NOTE 4
OPTICAL SECOND HARMONIC GENERATION FOR IN-PLANE POLARIZATION MAPPING (SHG) 16

SUPPLEMENTARY NOTE 5
NITROGEN VACANCY MAGNETOMETRY 25

SUPPLEMENTARY NOTE 6
SPIN CYCLOID WAVEVECTOR AND POLARIZATION RELATION IN POLED La-BiFeO_3 31

SUPPLEMENTARY NOTE 7
THERMALY EXCITED NON-LOCAL MAGNON TRANSPORT 37

References 43

SUPPLEMENTARY NOTE 1

X-RAY DIFFRACTION

The ferroelectric anisotropy in $Bi_{0.85}L_{0.15}FeO_3$ is expected due to the strain state of the orthorhombic substrate as a result of its monoclinic distortion. The representative θ−2θ x-ray diffraction pattern of $Bi_{0.85}L_{0.15}FeO_3$(80nm) is shown in Supplementary Figure 1. The observation of only the $00l$ peaks validates the single phase epitaxial growth on $DyScO_3$ $(110)_O$. The misfit strain between $Bi_{0.85}L_{0.15}FeO_3$ and $DyScO_3$ $(110)_O$ is ∼0.2% and ∼0.33% along the $[001]_O$ and $[1\bar{1}0]_O$, respectively. These values are evaluated using lattice constants measured using a reciprocal space map (RSM). $DyScO_3$ has an orthorhombic structure (space group $Pbnm$, lattice constants, a_0=5.440 Å, b_0= 5.717 Å, and c_0=7.903 Å). For $[110]_O$ substrate, the orthorhombic unit cell can be related to the tilted pseudocubic (monoclinic) unit cell through the following steps, a=c_0/2=3.952 Å. The difference between the substrate lattice constant is ∼0.1% and between the angles is 3%. In total, there is a large possibility of strain effect from the lattice difference as well as the monoclinic distortion of the substrate itself. We also identify the similar behavior in $Pb_{1-x}Sr_xTiO_3$ thin films deposited on $DyScO_3$ substrate [1]. A similar effect from the substrate is being used to create the anisotropic vortex tubes $[PbTiO_3/SrTiO_3]_n$ superlattices [2]. Therefore, anisotropy (from the substrate) is expected in the ferroelectric domain wall motion vis-à-vis magnon transport under in-plane electric field excitation. The directions of $Bi_{0.85}L_{0.15}FeO_3$, $DyScO_3$, and how the metal electrodes landed on the top where the condition of switchable and non-switchable is depicted below.

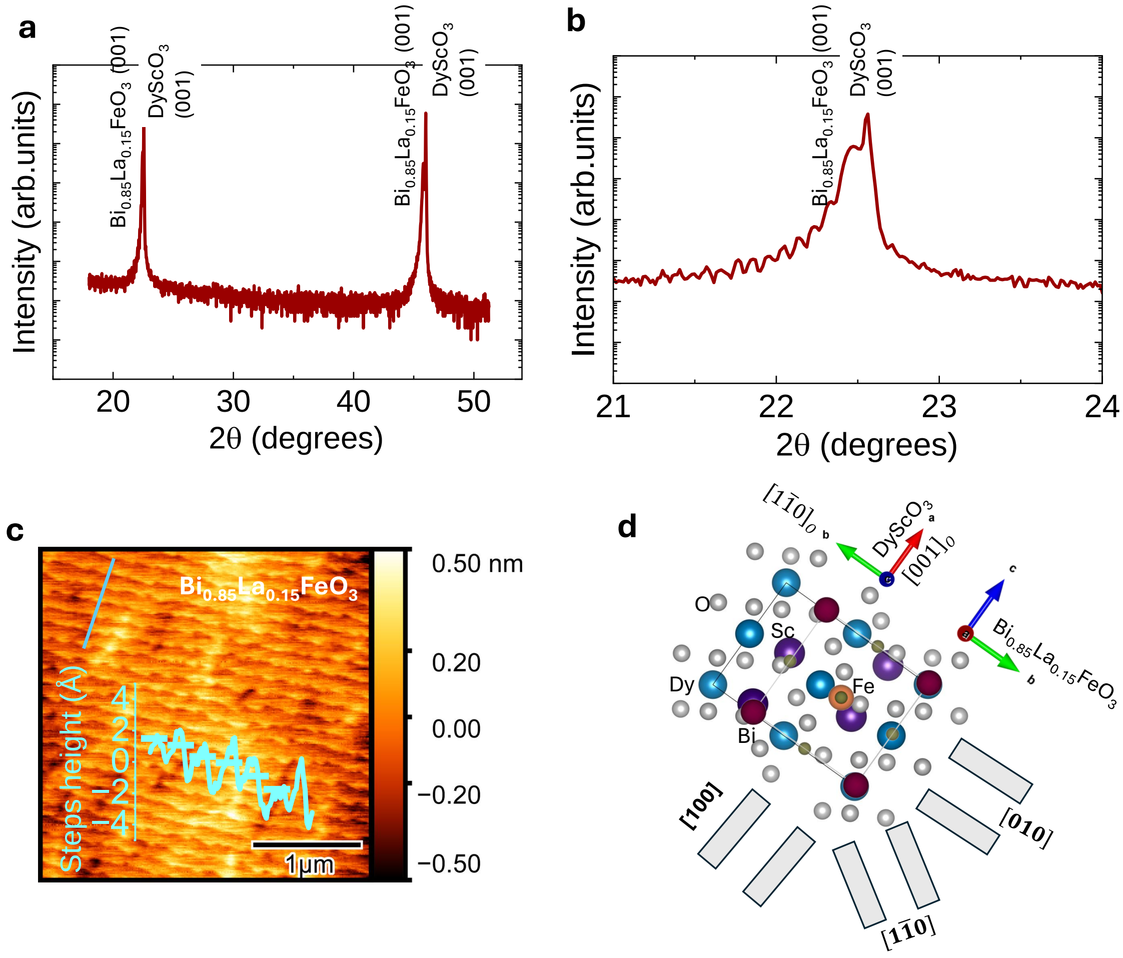

Supplementary Figure 1. **a** X-ray diffraction pattern of $Bi_{0.85}L_{0.15}FeO_3$.**b** Zoomed scan around $Bi_{0.85}La_{0.15}FeO_3$ [001] peak. Oscillations in the $Bi_{0.85}L_{0.15}FeO_3$ peaks indicate good-quality epitaxy growth. **c** Atomic force microscopy image of $Bi_{0.85}L_{0.15}FeO_3$ surface where the atomic terrace (inset plot) indicates the layer-by-layer growth. **d** Schematic of $Bi_{0.85}L_{0.15}FeO_3$ unit cell (drawn using VESTA), $DyScO_3$ substrate along with the metal electrodes aligned at different angles [100], [010] and [1$\bar{1}$0] pseudocubic directions with respect to the substrate. The [010] device electrodes are aligned with [1$\bar{1}$0] to the $DyScO_3$ orthorhombic notations.

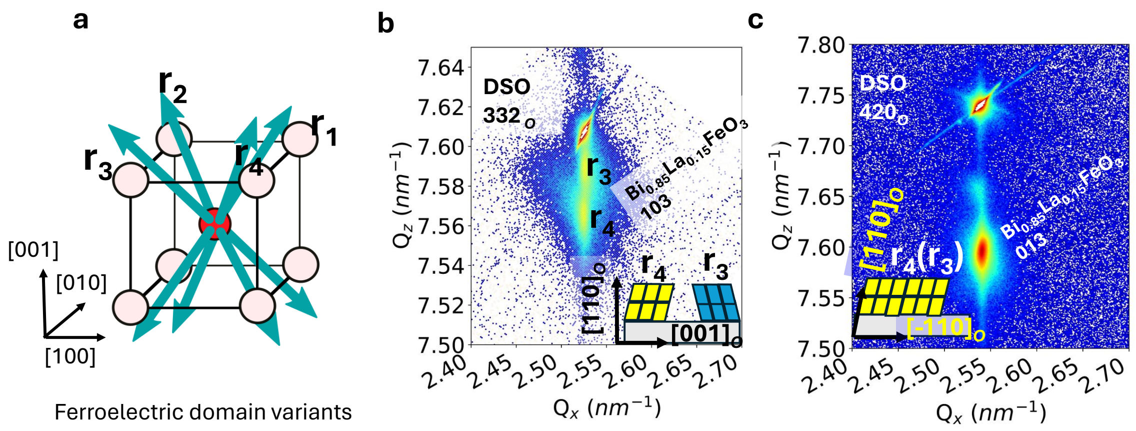

Supplementary Figure 2. **Reciprocal mapping of $Bi_{0.85}La_{0.15}FeO_3$:** a Schematic of Ferroelectric domain variant r_1, r_2, r_3, r_4. Asymmetric reciprocal space maps were recorded on $Bi_{0.85}La_{0.15}FeO_3$ thin films of 90 nm. Reciprocal space mapping of $(332)_O$ planes in DSO shows the (103) planes in $Bi_{0.85}La_{0.15}FeO_3$, suggesting that the $Bi_{0.85}La_{0.15}FeO_3$ is epitaxially strained to the DSO substrate. Using RSM, the in-plane (out-of-plane) lattice parameters of $Bi_{0.85}La_{0.15}FeO_3$ were found to be 3.960 (3.965 Å). The peak splitting occurs in the *h0l*-diffraction condition along (103), but not in the *0kl*-diffraction condition (013). This indicates that only two structural variants are present in these films grown on (220) $DyScO_3$ substrates, consistent with the PFM results in previous studies [3]. The two peaks in 103 reflections represent the *R*-like phase with the M_A structure, which is in agreement with previous reports [**?**]. A schematic illustration of the two-domain motif, as viewed along the $[1\bar{1}0]_O$ and $[001]_O$. Because the substrate has a monoclinic distortion along the $[0\bar{1}1]$ ($[010]_O$), only two structural variants r_3 and r_4, which have a spontaneous shear distortion along $[\bar{1}\bar{1}1]$ and $[\bar{1}1]$, respectively give rise to a net shear distortion along the monoclinic distortion of the substrate. Therefore, these two variants r_3 and r_4 are energetically favorable when the rhombohedral films are grown on $[110]_O$ substrates, in order to follow the substrate monoclinic distortion.

SUPPLEMENTARY NOTE 2

CROSS-SECTIONAL MICROSCOPY IMAGING AND POLARIZATION MAPPING

The polarization direction in $BiFeO_3$ is well understood which is along $< 111 >$ can choose either [111] [-111] [1-11] [11-1] [-1-11] [-11-1] [1-1-1] [-1-1-1]. From the atomic imaging, the projected atomic displacement (Methods, main text) is calculated to be 34.4±4.0 pm. The polarization vector direction is found to be 43.6°±3.8°. Thus in our case, the polarization of $BiFeO_3$ (Supplementary Figure 3) indeed is found set to be along [-1-11] or [1-11] as measured by the angle of polarization (∼45°). The atomic displacement (real) using the projected displacement and considering the pseudocubic unit cell is calculated to be $\frac{34.4}{\sqrt{2}\times\sqrt{3}} = 14.04\pm1.5\ pm$.

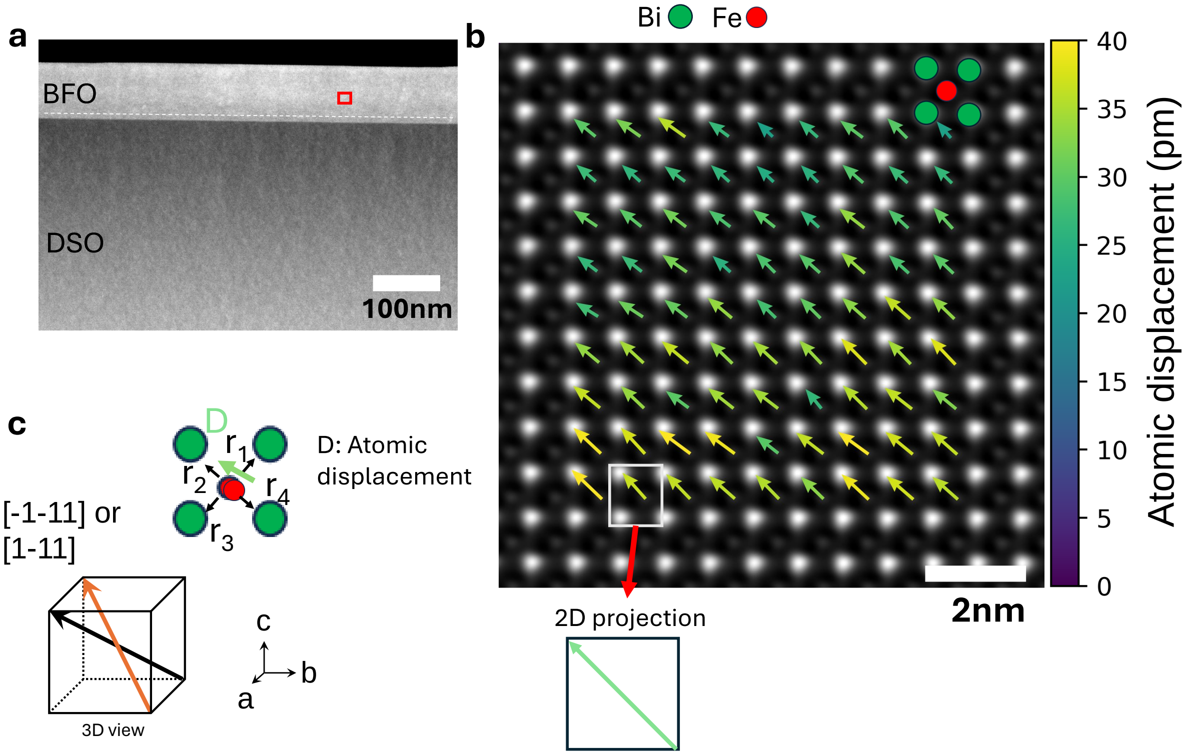

Supplementary Figure 3. Cross-sectional microscopy and the polarization mapping of $BiFeO_3$. **a** Low magnification TEM image of the $BiFeO_3$/DSO. The high-resolution image **b** shows the polarization vector mapping. **c** Scheme to evaluate the polar distortion using atomic displacement. TEM gives the 2D projection however the magnitude of the displacement vector provides the direction of the polar distortion in the unit cell.

In the La substituted $BiFeO_3$, the polarization is shifted away from $< 111 >$ and relaxed along $< 112 >$ [3] and (Figure 1, theory, main text). From the STEM imaging (Supplementary Figure 3), the projected atomic displacement is found to be 32.4±3.5 pm, and using the pseudocubic unit the real atomic displacement is calculated to be $\frac{32.4}{\sqrt{5}\times\sqrt{6}} = 5.9\pm0.4\ pm$, which is smaller than the $BiFeO_3$ (discussed above) indicating the modifying polar distortion upon La-substitution. The polarization angle is evaluated to be 63.6±2.8° ∼63°. The 2D projection revealed the P-vector is downward choosing the [-1-1-2] or [1-1-2]. The magnitude and the direction confirm the polarization orientation is indeed along [112] in $Bi_{0.85}L_{0.15}FeO_3$.

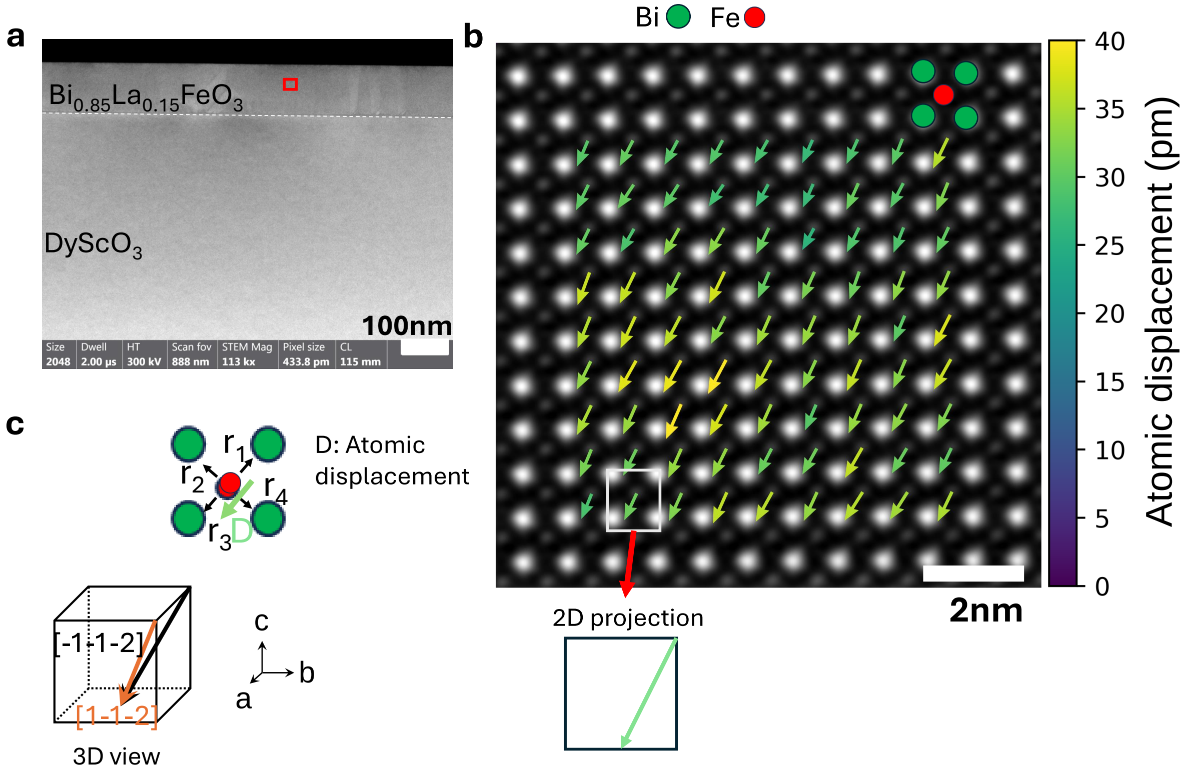

Supplementary Figure 4. Cross-sectional microscopy and the polarization mapping of $Bi_{0.85}L_{0.15}FeO_3$. **a** Low magnification TEM image of the $Bi_{0.85}L_{0.15}FeO_3$/DSO. High resolution **b** shows the polarization vector mapping. **c** Scheme to evaluate the polar distortion using atomic displacement. TEM gives the 2D projection however the magnitude of the displacement vector provides the direction of the polar distortion in the unit cell.

SUPPLEMENTARY NOTE 3

FERROELECTRIC DOMAIN CHARACTERISTICS

Piezoresponse Force Microscopy (PFM), is a scanning probe technique that leverages the inverse piezoelectric effect, elucidating the relationship between mechanical deformation in materials subjected to an applied electric field. In a typical Atomic Force Microscopy configuration, a minor AC voltage is applied between a conductive tip (here Pt) and a piezoelectric material (here $Bi_{0.85}L_{0.15}FeO_3$). This induces a local piezoelectric vibration, leading to the oscillatory deflection of the material surface. This deflection can be detected by the tip and transmitted to the lock-in system. The resulting amplitude signal provides information about the magnitude of the piezoelectric coefficient, while the phase signal identifies the polarization direction in the ferroelectric sample. PFM imaging unveils intrinsic ferroelectric domain structures by showcasing variations in spontaneous polarization across different domains through distinct PFM amplitude and phase signals.

To detect the polarization component oriented perpendicular to the sample surface, the vertical or out-of-plane (OP) PFM mode is employed. The tip, in contact with the sample surface, reflects the local piezoelectric response to the first harmonic component of tip deflection. The phase (ϕ) provides insights into the local polarization directions beneath the tip.

For detecting the polarization component within the plane of the sample surface, the lateral or in-plane (IP) PFM signal is recorded. The working principle of the lateral mode is grounded in a bias-induced surface shearing effect, with the detected polarization component sensitive to the direction perpendicular to the cantilever axis. The difference between the OP and IP modes is set up by choosing the desired oscillating frequency. For the OP case, the frequency of the cantilever is about 350kHz, and respectively for the in-plane is about 650kHz. Determining the

two in-plane components of the polarization vector involves imaging the same region before and after a φ =90° rotation. The integration of an out-of-plane (OP) and two orthogonal in-plane (IP) PFM modes constitute the vector PFM approach. This combined methodology effectively reveals the three-dimensional contributions to the polarization vector in specific regions of a material.

Using the approach described above, one can easily distinguish the ferroelectric domain variant as depicted in Supplementary Figure 5a. Individual domains have net polarization either IP or OP depending on the crystal structure. In the case of $Bi_{0.85}L_{0.15}FeO_3$ (used in this work), both IP and OP polarization are expected due to the rhombohedral (monoclinic) phase and polarization direction along $[111]$ or $[112]$ direction. Since the polarization is in diagonal to the unit cell, the in-plane domains are expected to be significant. Let's go back to the domain variants in the $Bi_{0.85}L_{0.15}FeO_3$, which depend on the strain effect of the substrate. These variants are possibly in three types four, two, and single variants where each variant constitutes one domain. To probe the type of variant the sample can be rotated within the film plane (Supplementary Figure 5a, bottom panel) and record the PFM signal. It shows how during the rotation, one can map out the polarization direction and hence number of variants.

Supplementary Figure 5 b and c, reveals the domain switched by $90°$ when the sample is rotated by $\phi = 90°$. This is further substantiated in different devices as shown in Supplementary Figure 7. This also shows the single variant in between the electrodes and two variants in the pristine state. How the two variants go into a single variant or single domain is demonstrated in Supplementary Figure 7 8 9 10 11. The polarization mapping is further discussed in the next section.

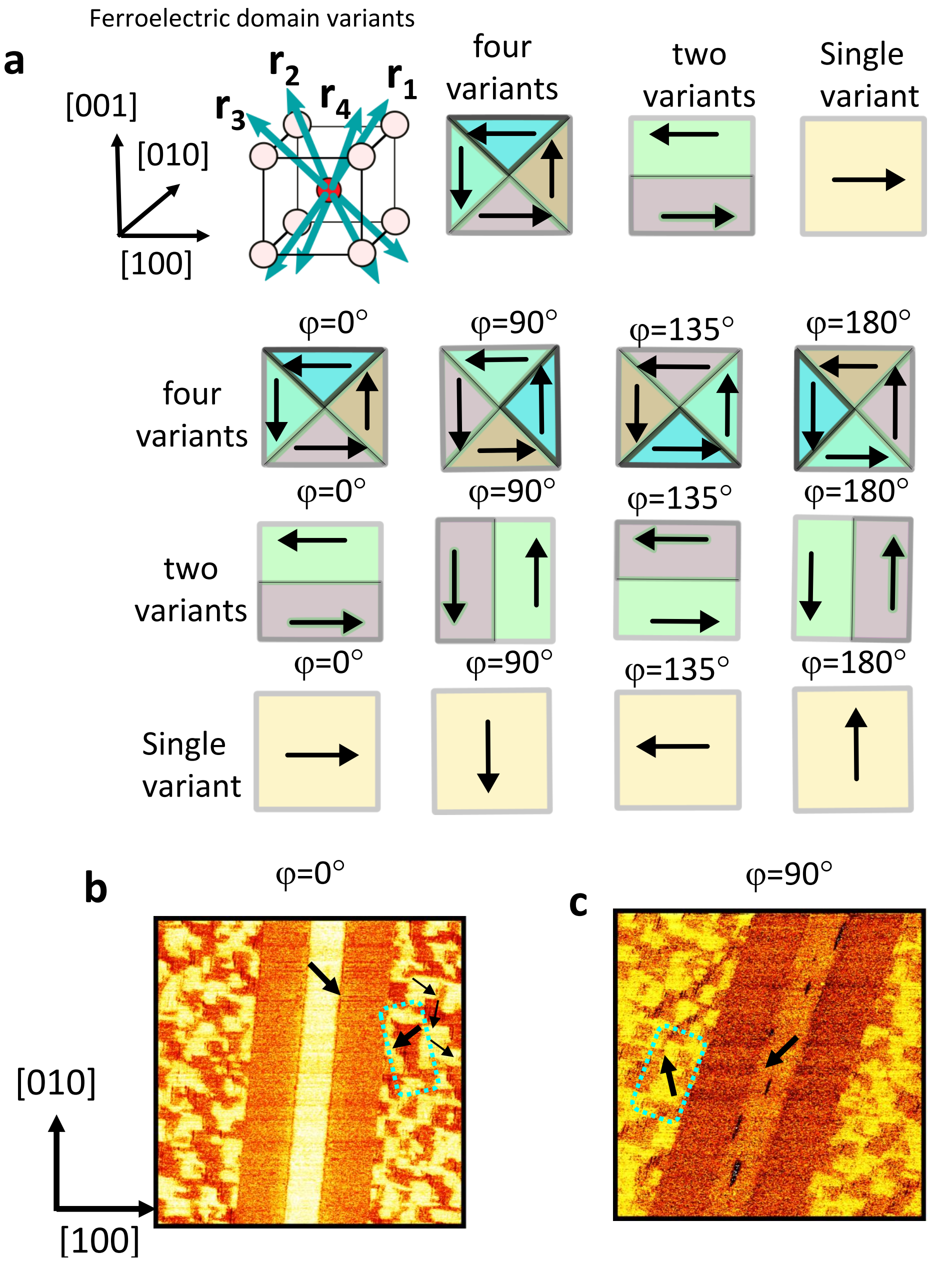

Supplementary Figure 5. Ferroelectric domain variants and PFM imaging. a $Bi_{0.85}L_{0.15}FeO_3$ unit cell and the four possible polarization variant r_1, r_2, r_3 and r_4. There are three possible situations in $BiFeO_3$ compounds to be presented as four, two, and a single variant based on the ferroelastic domains. In four variants, four, 90° domain walls are present, two variants most likely form 180° domain wall whereas a single variant is free from the ferroelectric domain wall. The bottom panel in **a**, depicts the domain variants under in-plane rotation of the sample or the domain of different kinds. **b** PFM images after poling and measured at $\varphi = 0°$ and $\varphi = 90°$ physically rotated sample. It appears that the polarization is rotated by $\varphi = 90°$ indicative of the two variant systems under consideration.

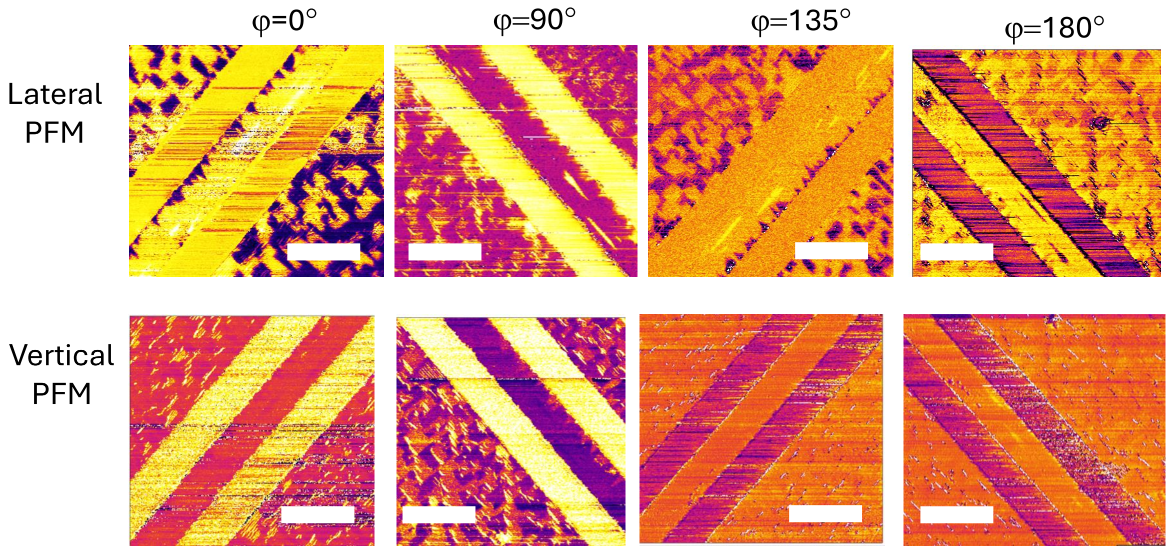

Supplementary Figure 6. **Lateral and Vertical PFM:** PFM phase maps of [010] poled device recorded after physically rotated by 90° and Lateral and vertical PFM images were recorded. A single-color contrast in all directions as well as in both PFM geometries indicates the single ferroelectric domain formation. The scale bar is 3μm.

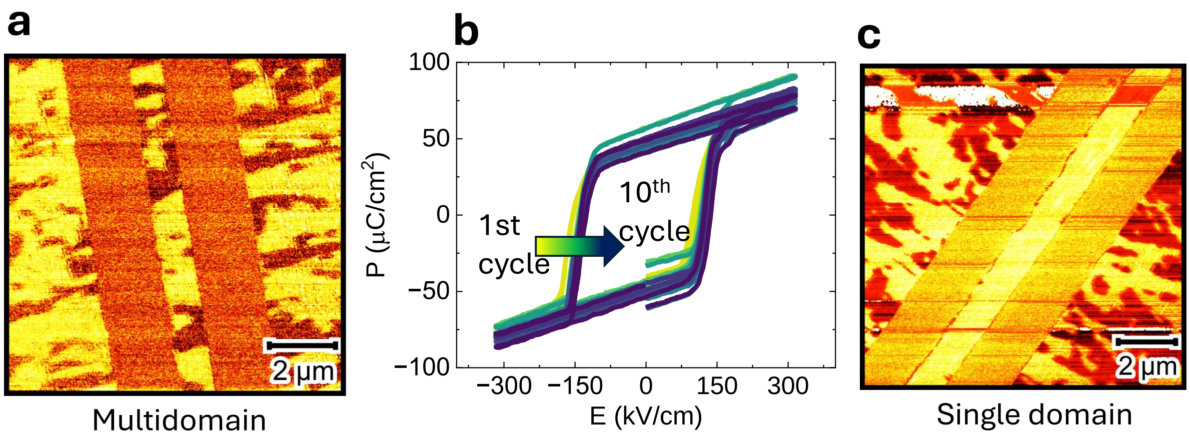

Supplementary Figure 7. **The evolution of single domain:** **a** PFM image of the pristine (unpoled) state of device [010]. **b** Ferroelectric hysteresis is recorded from 1-to-10 cycles and the evolution from multidomain to a single domain is represented by the squareness of the hysteresis evolution. **c** Corresponding PFM images of the single domain after poling.

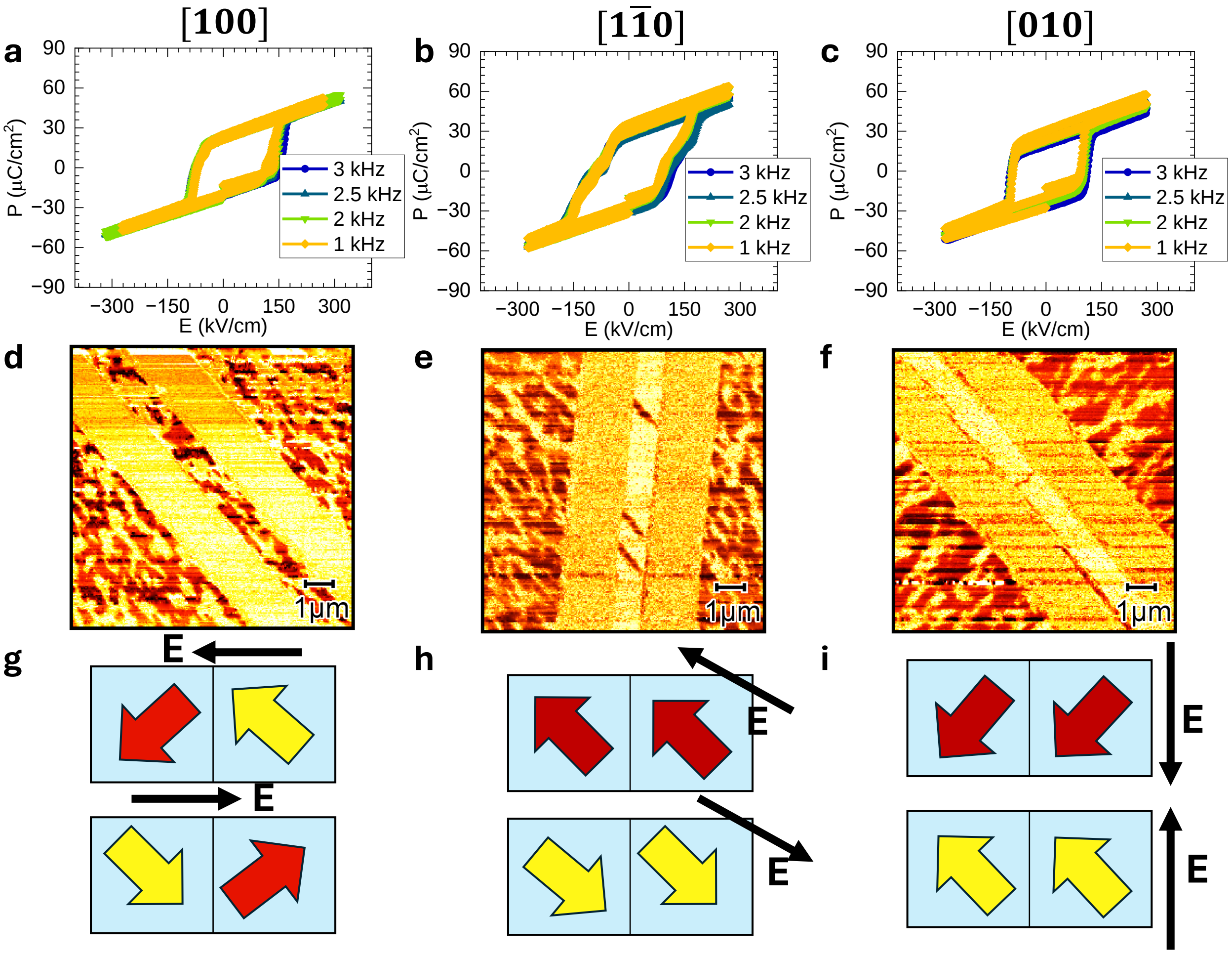

Supplementary Figure 8. The evolution of hysteresis in the device with multidomain and single domain devices: Ferroelectric hysteresis in the device **a** [100], **b** [1$\bar{1}$0] and **c** [010]. In the case of [100], the ferroelectric state remains multidomain **(d)** whereas other devices form a single domain upon poling **(e) and (f)**. The ferroelectric polarization hysteresis has different features such as remnant polarization is lowest in [100] and highest in [1$\bar{1}$0]. This is correctly followed by the rule to $P_{[1\bar{1}0]} = \sqrt{2}P_{[100] or [010]}$. Depending on the external electric field, the domain re-orientation is presented in **g** [100], **h**[1$\bar{1}$0] **and i** [010]. The domain wall motion leads to the formation of a single domain in two angles when the two domain polar vectors are parallel.

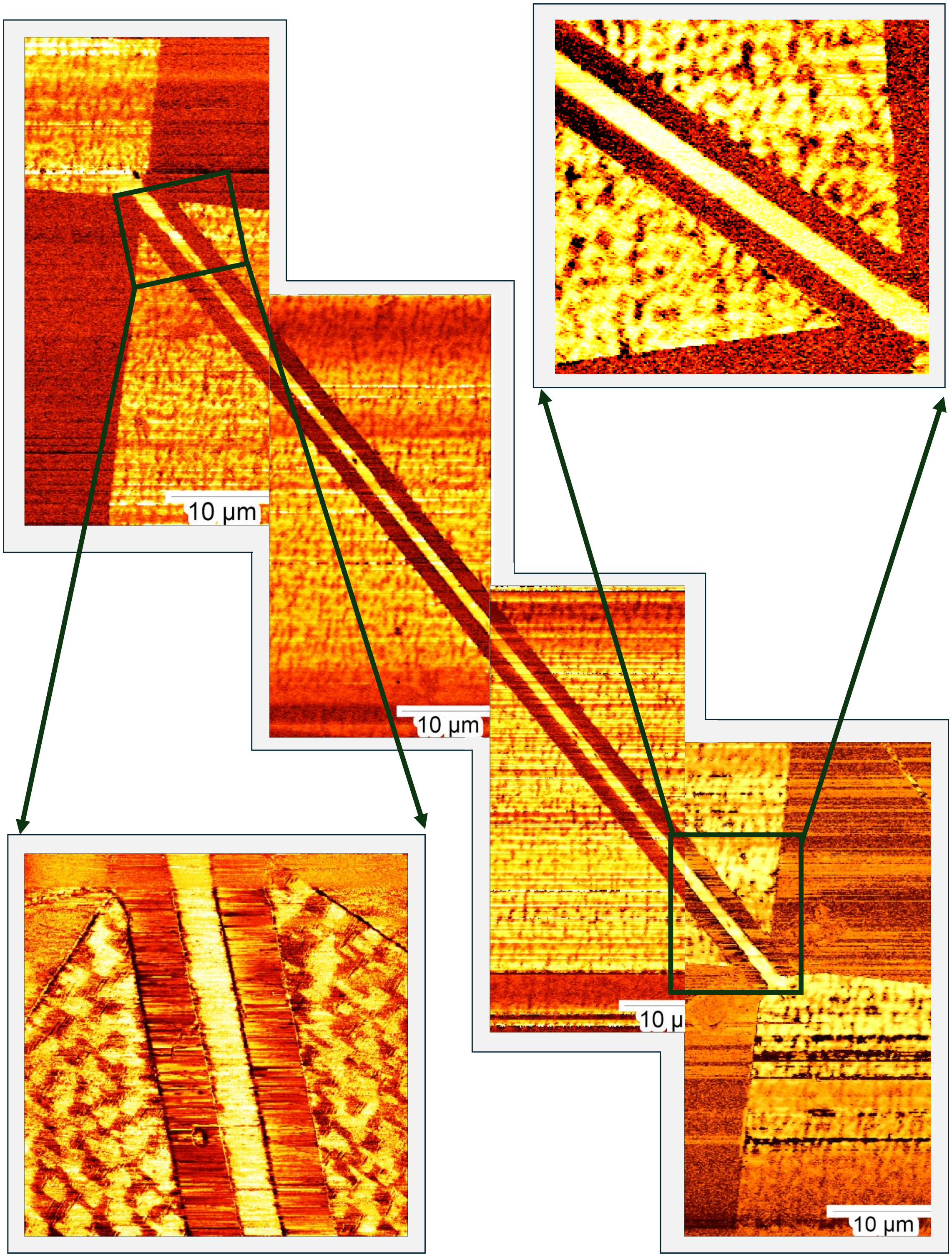

Supplementary Figure 9. **Single ferroelectric domain:** Full device piezo force microscopy image to show a robust single domain formation after poling by the in-plane single electrical pulse (+150 kV/cm). The insets are the zoomed PFM scans to magnify the ferroelectric single domains in the two extreme edges of the 100μm long stripe of a nonlocal device. This PFM image belongs to one of [010] devices as depicted in Figure 2a (main text).

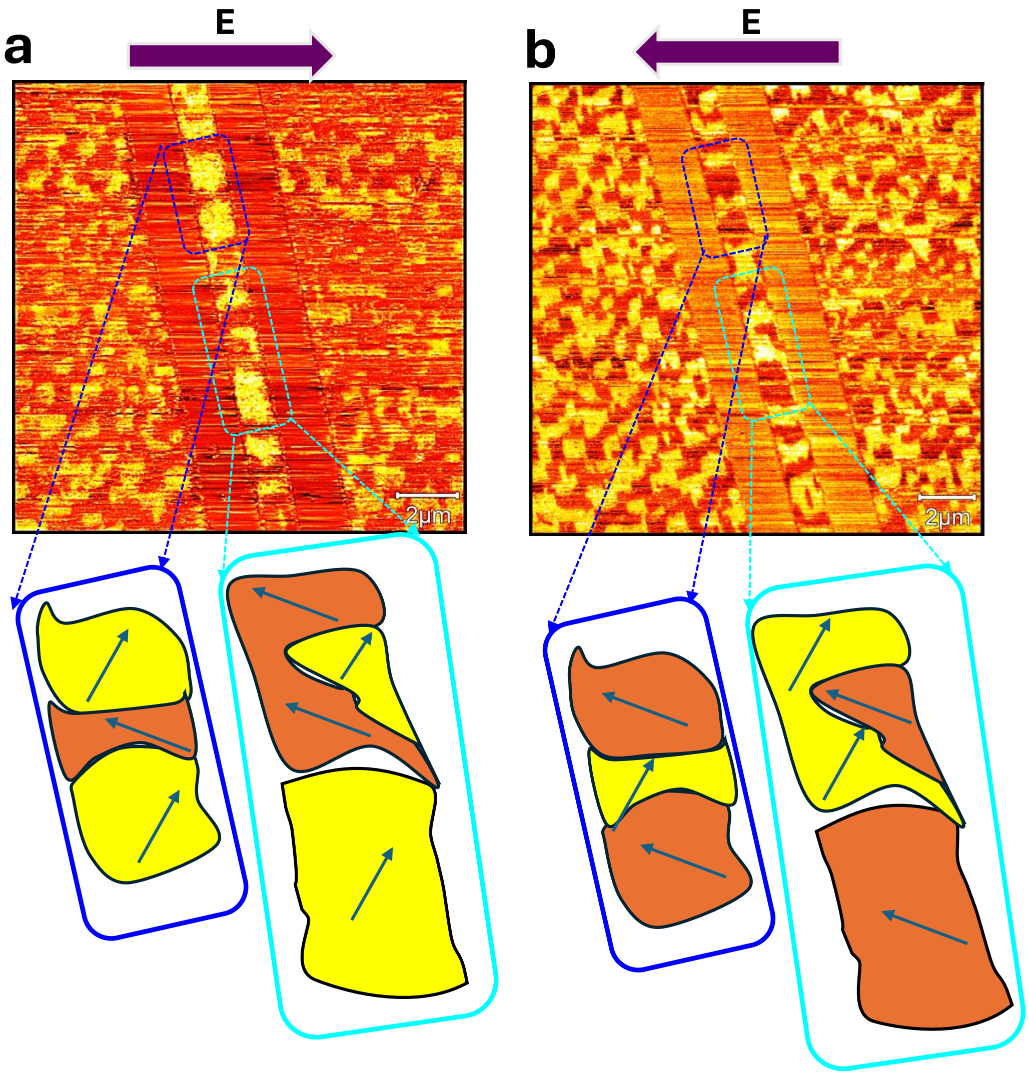

Supplementary Figure 10. **Piezoforce microscopy of** [100] **device**: PFM images [100] device after electrical (E) pulsing in two opposite directions. The big arrow indicates the field direction. Rectangles are used to identify the same area scanning in opposite poling. Rectangles within the metal stripes show the change of ferroelectric polarization locally as shown in zoomed schematics. Due to the non-regular domain pattern, the domains have some sections of head-to-head or tail-to-tail situations which create out-of-phase situations discussed in the next figure.

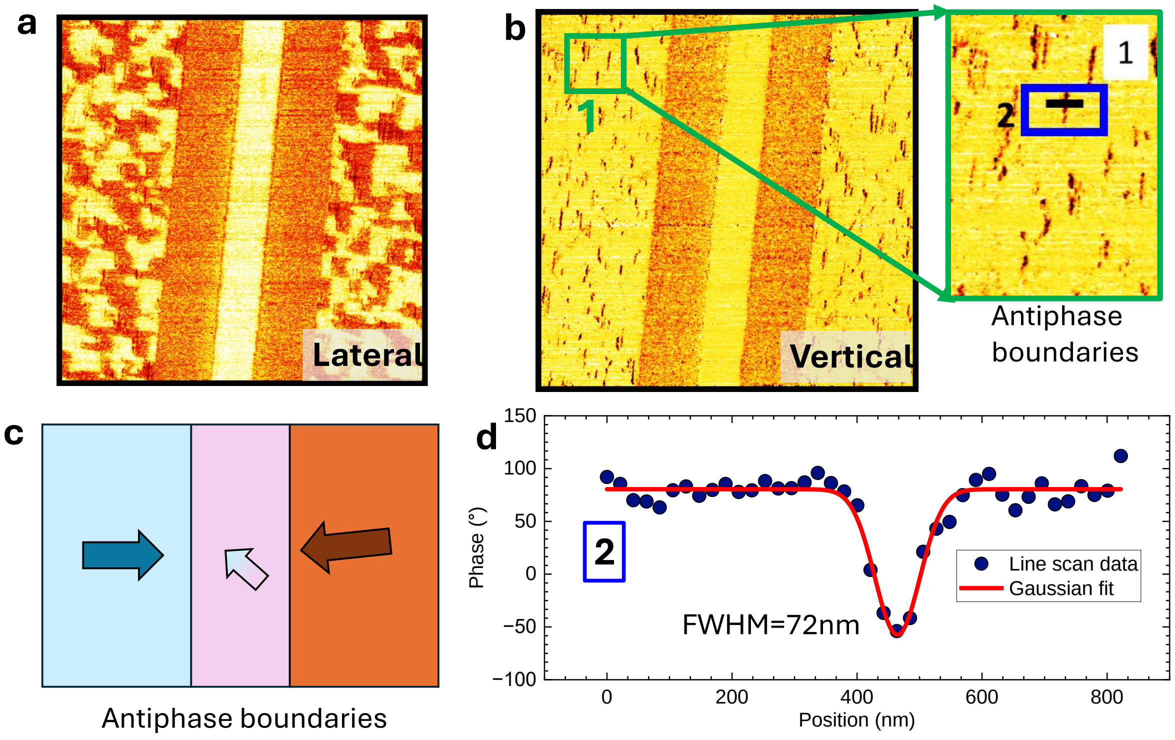

Supplementary Figure 11. **Antiphase boundaries in multidomain regions**: **a** Lateral and **b** vertical PFM images of [010] device after polling. The zoomed section of the vertical phase contrast represents the antiphase domain walls. **c,d** the antiphase DW and line scan shows the out-of-phase contrast around DWs.

SUPPLEMENTARY NOTE 4

OPTICAL SECOND HARMONIC GENERATION FOR IN-PLANE POLARIZATION MAPPING (SHG)

Optical Second Harmonic Generation (SHG) involves the process of doubling the frequency of an incident light wave. The emission of SHG in a crystal is contingent upon its point group symmetry, making SHG responsive to any deviation from point symmetry in the material. As the development of ferroic order is closely associated with a reduction in crystal point-group symmetry, SHG serves as an effective means to investigate such ordered states in ferroelectric materials. Notably, this technique proves valuable for probing coexisting ferroic states or multidomain domains or different variants as we discussed above, in the multiferroics. The macroscopic description of the source term for SHG is described through the polarizability tensor [4, 5],

$$P_i^{2\omega} = \epsilon_0 \chi_{ijk} E_j^{\omega} E_{kj}^{\omega} \tag{1}$$

where E^{ω} is the incident electric field (laser light) at frequency ω, $P_i^{2\omega}$ is the induced polarization in the nonlinear medium at frequency 2ω, which acts as a source of an emitted, frequency-doubled, light wave with intensity $I_{SHG} \propto |Pi^{2\omega}|$. ϵ_0 is the third-rank nonlinear susceptibility tensor, parameterizing the non-linear light-matter interaction. The indices i, j, k are the components along x, y, z as depicted in Supplementary Figure 12a. The form of the χ_{ijk} tensor is dictated by the specific crystal point group symmetry. The nonlinear polarization at 2ω radiates back electrical field,

$$\Delta^2 E^{2\omega} - \epsilon_0 \mu \frac{\partial^2 (E^{2\omega})}{\partial t^2} = \mu \frac{\partial^2 (P^{2\omega})}{\partial t^2}. \tag{2}$$

The corresponding intensity will be,

$$I^{2\omega} = \frac{1}{2}\epsilon_0 \nu (E^{2\omega})^2. \tag{3}$$

Experimentally, one can access the specific χ_{ijk} components by carefully selecting incident and detected light polarization or the angle of polarization by selecting φ. The spectral variation of the $\chi(2)$ tensor can be determined using a tunable wavelength light source, offering detailed insights into particular electronic transitions and optimizing second harmonic generation response. Figure 12a illustrates a basic SHG setup being used in transmission mode in this work. The probe beam's polarization direction is defined by the polarizer angle, while the detected SHG light's polarization direction aligns with the analyzer angle. Since the second harmonic light is spectrally distinct from the fundamental light, monitoring the SHG frequency separately from the probe beam intensity simplifies the process, making it a background-free characterization technique.

In the context of ferroelectrics, the breaking of inversion symmetry is facilitated by a polar distortion, resulting in non-zero χ_{ijk} components. To comprehensively characterize this polar distortion along a specific crystallographic direction and distinguish between various tensor components, one can employ polarizer measurements. In these measurements, the polarization direction of the second harmonic light (analyzer angle) is fixed, while the polarization of the probe beam is systematically rotated.

During the experiment, it is crucial to account for three distinct coordinate systems and their interrelations: (i) the lab coordinate axes (x, y, z), which define the coordinate system within the optical setup; (ii) the sample coordinate axes (X, Y, Z), detailing the orientation of the sample edges in relation to the optics; and (iii) the crystal physics axes (X_1, X_2, X_3), representing the orientation of the nonlinear optical tensor coordinates for each domain variant

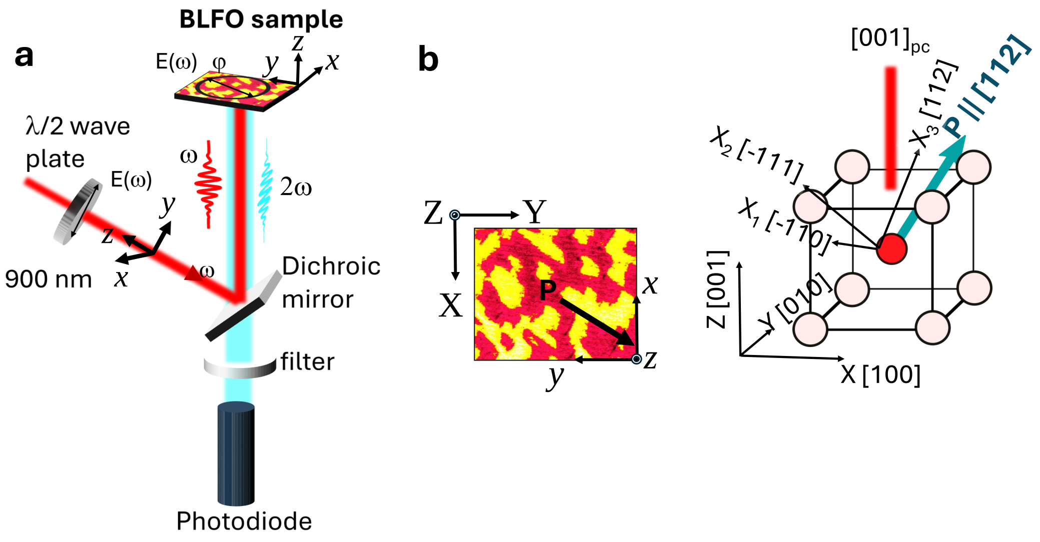

Supplementary Figure 12. **Second Harmonic Generation (SHG): a** Typical SHG measurement setup in transmission. A laser (wavelength, 900nm) is used to excite the linear dichroic signal and harmonic signal detected by the photodiode detector. The incident probe beam is linearly polarized by a Glan-Taylor prism. The polarization of the probe beam is then set to an arbitrary polarization state by a rotatable half-wave plate. A half-wave plate for linear polarization states or a quarter-wave plate to achieve circular polarization. A focusing lens controls the spot size on the sample. The low-pass filter removes any second harmonic light generated in the polarization optics before the sample. The fundamental beam and higher-order harmonics are blocked by band/high-pass and low-pass filters. The SHG light is collected by the lens, and the polarization state is analyzed by projection on a second rotatable GT prism. The SHG intensity is integrated after passing a monochromator and photomultiplier tube. **b** Schematics illustration of the axes orientation and polarization direction in the $Bi_{0.85}La_{0.15}FeO_3$ thin film sample. The transformation relations between the crystal physics axes (X_1, X_2, X_3) and the sample coordinate axes (X, Y, Z). The crystal physics axes (X_1, X_2, X_3) were determined from the $3m$ point group symmetry. **P** is in parallel to the [112]pc direction, as well as the X_3 of the crystal physics axes. In the SHG experiment, the fundamental light was incident normally onto the (001)pc surface as shown by red shaded line.

(Supplementary Figure 12b). Equation 3 encompasses polarization contribution directions aligned with the crystal physics axes of the $3m$ point group symmetry. The linear polarization of the incident beam is manipulated using a half-wave plate affixed to a motorized rotation stage. This allows precise control over the azimuth angle, φ, with respect to the x-axis in the $x-y$ plane of the (x, y, z) coordinate system. Consequently, the electric field of the fundamental light, expressed as $E^{\omega}(\varphi) = (E_X, E_Y, E_Z) = (-E_x, -E_y, E_z)= (-E_0\cos\varphi, -E_0\sin\varphi, 0)$, can be

accurately rotated. Here, E_1, E_2, E_3 are employed to denote the components of $E^{\omega}(\varphi)$ in the crystal physics axes ($X_1 \parallel [-110]$, $X_2 \parallel [-1-11]$, $X_3 \parallel [112]$). The values of φ determine specific light polarization states, allowing the determination of these components under varying conditions to enable the polarization measurement of different domains.

The light-induced non-linear polarization of the polarized domains can be described using the $3m$ point group-based SHG tensor in a relation derived from Equation 1,

$$\begin{pmatrix} P_1 \\ P_2 \\ P_3 \end{pmatrix} = \begin{pmatrix} 0 & 0 & 0 & 0 & d_{31} & -d_{22} \\ -d_{22} & d_{22} & 0 & d_{31} & 0 & 0 \\ d_{31} & d_{31} & d_{31} & 0 & 0 & 0 \end{pmatrix} \begin{pmatrix} E_{12} \\ E_{22} \\ E_{32} \\ E_{32} \\ 2E_2E_3 \\ 2E_1E_3 \\ 2E_1E_2 \end{pmatrix} \tag{4}$$

where d_{ij} ($i, j =$ 1,2,3) is the reduced nonlinear susceptibility tensor. The (P_1, P_2, P_3) corresponds to the polarization of SHG light (2ω), whereas the (E_1, E_2, E_3) corresponds to the electrical field of fundamental light (ω). By analyzing the fundamental light polarization settings, sample orientation, and nonlinear susceptibility tensors, we can derive valuable insights into intrinsic sample properties. These include the distribution of ferroelectric domains, as discussed in the main text and here, along with information about orientation and local structural symmetry. The

polarization components can be solved from the Equation 4 and written as,

$$P_1^{2\omega} = 2d_{13}E_1E_3 - 2d_{22}E_1E_2$$

$$P_2^{2\omega} = -d_{22}E_1^2 + d_{22}E_2^2 + 2d_{31}E_2E_3$$

$$P_3^{2\omega} = d_{31}E_1^2 + d_{31}E_2^1 + d_{33}E_3^2$$

The SHG intensity of polarization can be controlled by the incident light through the half-wave plate by selecting the angle φ. The SHG intensity achieved the maximum and the minimum at specific φ with a 90° difference, for example, 45° and 135°, respectively. Due to the symmetry of the $Bi_{0.85}La_{0.15}FeO_3$, the in-plane polarization component $P_{net} \parallel$ diagonal of the substrate surface at either 45° or 135° with respect to x-axis, thus we can determine the SHG polarization map using the two extreme cases in parallel and perpendicular to in-plane P_{net}. We chose the two angles φ =45° and φ =135° polarization states of the fundamental light to measure the net in-plane polarization within the oppositely poled devices. At φ =45° (light polarization $\parallel X_1$ or $[\bar{1}10]$), the electric field component would be $(E_1, E_2, E_3 = (E_0, 0, 0))$. And at φ =135° (light polarization $\perp X_1$ or $[\bar{1}10]$ and $\parallel$ $[\bar{1}\ \bar{1}\ 0]$), the electric field component would be $(E_1, E_2, E_3 = (0, \frac{\sqrt{3}}{3}E_0, -\frac{\sqrt{6}}{3}E_0))$. The intensity in two orthogonal directions would be $I_x^{2\omega} \propto (\mathbf{P}^{2\omega}.\mathbf{e}_x)^2$, $I_y^{2\omega} \propto (\mathbf{P}^{2\omega}.\mathbf{e}_y)^2$. And total intensity is given by

$$I_{SHG} = I_x^{2\omega} + I_y^{2\omega} \propto [\,|P_1^{2\omega}|^2 + |P_2^{2\omega}|^2 + |P_3^{2\omega}|^2]\,. \tag{5}$$

We noticed that the intensity is maximum/minimum at φ =45° or φ =135° (Supplementary Figure 13a and 13b). We evaluate the difference between the two orientations to measure the

real intensity from the polarization plotted in Supplementary Figure 13c. This is also called a linear dichroism. The polarization (P_{net}) intensity is maximum in the device [010] $\parallel$ φ =45° and hence P_{net} is made and angle 45° to metal electrodes (Supplementary Figure 13c, bottom) and no signal at φ =135°. This tends to explain the angle of polarization in the other devices after poling. For the domain variants, these results mean that SHG intensity would achieve higher values when the fundamental light polarization is parallel to the in-plane component of spontaneous polarization than when the fundamental light polarization is perpendicular to it. That is the reason why in these two cases the domain variants show a huge contrast in SHG mapping in the multidomain case (Supplementary Figure 13c device [100], top panel).

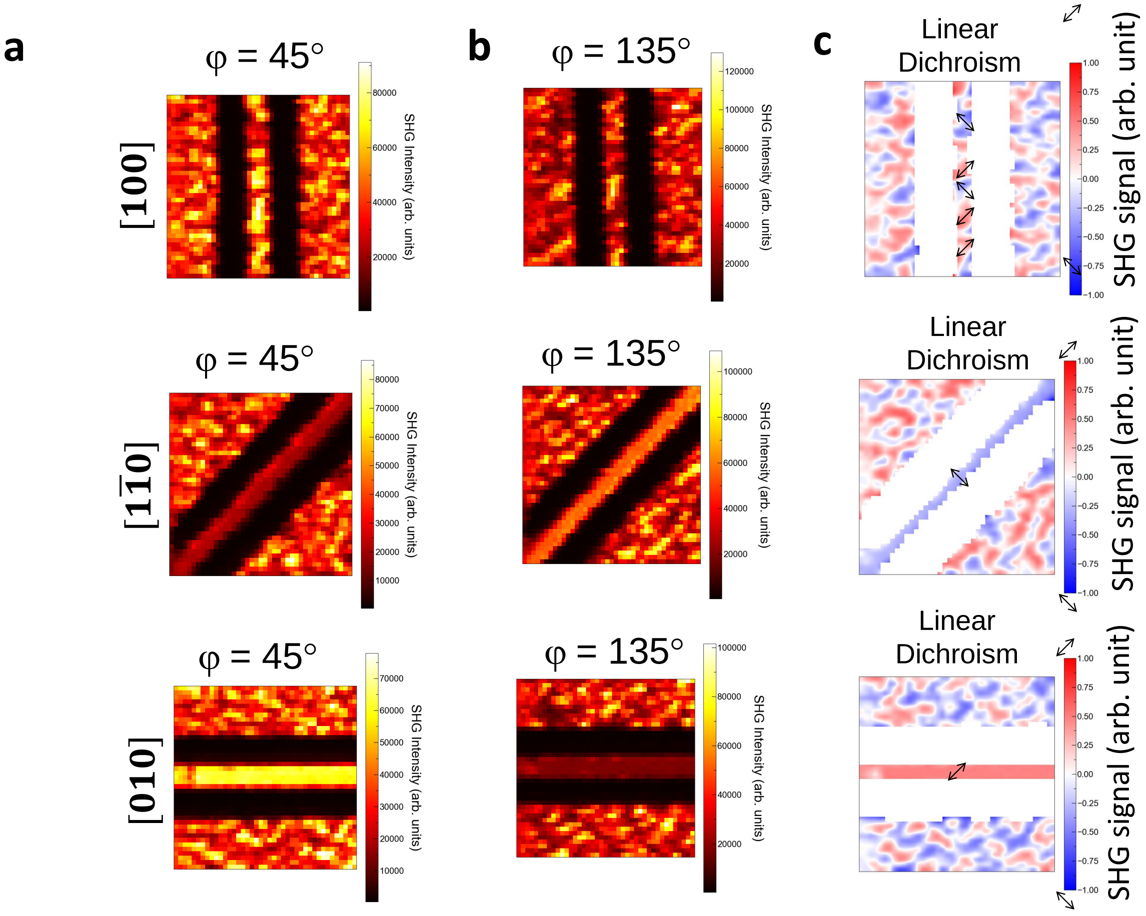

Supplementary Figure 13. **Second Harmonic Generation (SHG):** Polarization maps recorded at **a** φ=45° and **b** φ=135° in three device orientations [100], [010] and [1$\bar{1}$0], respectively top, middle and bottom panel. **c** Differential map between **a** and **b** , i.e., $[I(45) - I(135)]/[I(45) + I(135)]$.

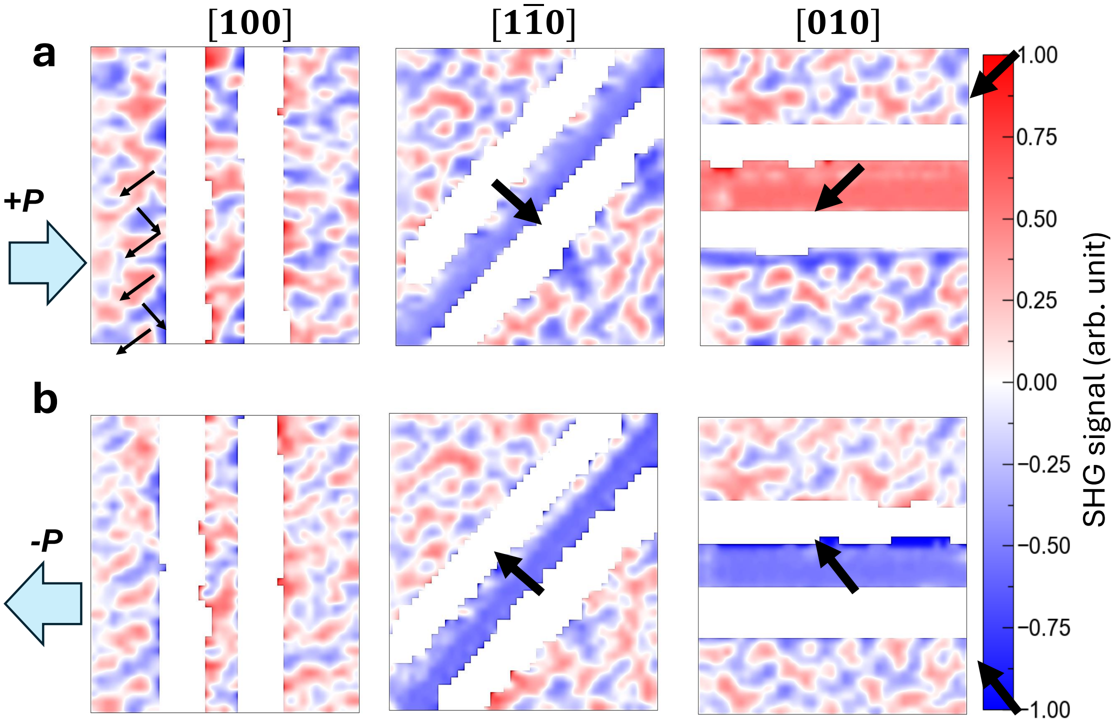

Supplementary Figure 14. **Second Harmonic Generation (SHG): a b** Polar map recorded on oppositely poled devices. Arrows represent the direction of the polarization in a single/multiple domain.

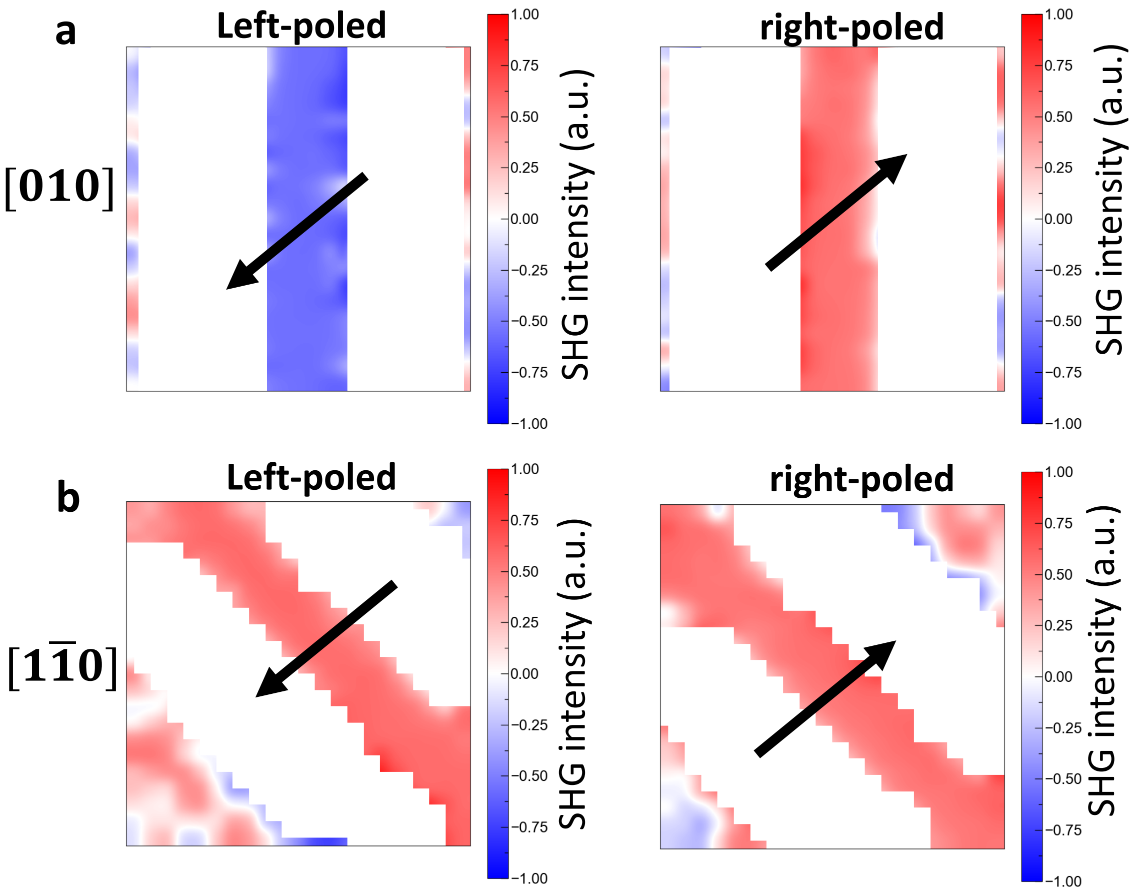

Supplementary Figure 15. **Second Harmonic Generation (SHG): a b** Polar map recorded on oppositely poled devices. Arrows represent the direction of the polarization in a single/multiple domain.

SUPPLEMENTARY NOTE 5

NITROGEN VACANCY MAGNETOMETRY

Scanning nitrogen-vacancy (NV) magnetometry allows us to quantitatively detect the stray magnetic field from the very weak magnet such as compensated ferromagnet or antiferromagnetic thin layers [6, 7] such as $BiFeO_3$ [8, 9]. NV center defect comprises a nitrogen atom (N) substituting for a carbon atom and a vacancy (V) in one of the nearest neighboring sites within the diamond crystal lattice (Supplementary Figure 16a). This state features a spin triplet (^{3}A) ground level that can be initialized as depicted in Figure 16b, coherently manipulated, and read out solely through optical means at room temperature.

An integral characteristic of the NV defect pertains to its fundamental attribute: the ground state exists as a spin triplet state labeled $^3A_2$. This state's sub-levels undergo energy division due to spin–spin interaction, resulting in a singlet state with spin projection $m_s = 0$ and a doublet with $m_s = \pm 1$. In the absence of a magnetic field ($B = 0$), these $m_s = \pm 1$ states are degenerate. The spin projection m_s denotes the alignment along the intrinsic quantization axis of the NV defect, aligned with the crystal axis ($[111]$) joining nitrogen and the vacancy. Microwave excitation can be used to couple the $m_s = 0$ and $m_s = \pm 1$ states, allowing coherent manipulation of the spins. Optical excitation of the defect occurs through spin-conserving transitions to a spin triplet ^3E excited level. The ^3E level shares the same quantization axis and gyromagnetic ratio as the ground level. Upon excitation to the ^3E level, the NV defect can relax through either a radiative transition, resulting in broadband red photoluminescence (PL), or a non-radiative inter-system crossing to singlet states (^1A and ^1E). These singlet states significantly influence the spin dynamics of the NV defect. Optical transitions, predominantly spin conserving ($m_s = 1$), coexist with non-radiative spin selective inter-system crossing to the ^1E singlet state, which

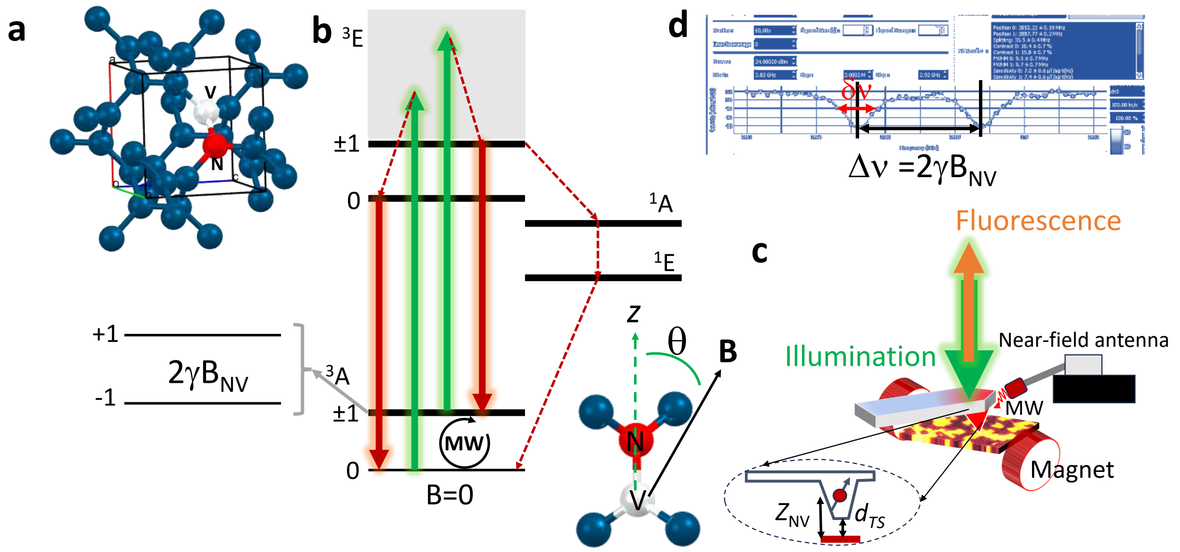

Supplementary Figure 16. Nitrogen–vacancy (NV) centers in diamond. (a) An NV center is formed by a substitutional nitrogen atom and an adjacent vacancy in the diamond lattice. (b) The energy levels and optical transitions of the NV electron spin. The spin states can be polarized with a green laser and read out by fluorescence intensity. (c) Ground states of an NV center under an external magnetic field. The degeneration of states is lifted by the Zeeman effect. The hyperfine interaction with surrounding ^{13}C nuclear spins brings an inhomogeneous broadening. The magnetic field is measured at around $\theta = 53°$ from the NV axis. (d) Typical optical detected magnetic resonance spectrum of an NV center. The strength of the external magnetic field can be extracted from the resonant positions of the spectrum.

exhibits strong spin selectivity. The shelving rate from the $m_s = 0$ sub-level is notably smaller than that from $m_s = \pm 1$. Conversely, the NV defect tends to decay preferentially from the lowest $^1A_1$ singlet state to the ground state $m_s = 0$ sub-level. These spin-selective processes result in a non-thermal electron spin polarization into $m_s = 0$ through optical pumping. The photoluminescence intensity of the NV defect is significantly higher for $m_s = 0$ state is populated. Such a spin-dependent PL response enables the detection of ESR (electron spin resonance) on a single defect by optical means. Indeed, when a single NV defect, initially prepared in the $m_s = 0$ state through optical pumping, is driven to the $m_s = \pm 1$ spin state by applying a resonant microwave (MW) field, a drop in the PL signal is observed. For magnetometry, the principle of the measurement is similar to the one used in optical magnetometers based on the precession

of spin-polarized atomic gases. The applied magnetic field is evaluated through the detection of Zeeman shifts of the NV defect electron spin sub-levels. Indeed, when a magnetic field is applied in the vicinity of the NV defect (Supplementary Figure 16c), the degeneracy of $m_s = \pm 1$ spin sub-levels is lifted by the Zeeman effect, leading to the appearance of two resonance lines in the ESR spectrum (Supplementary Figure 16d). A single NV defect therefore behaves as a magnetic field sensor with an atomic-sized detection volume.

The magnetic field is imprinted into the spectral position as $\Delta\nu$ of NV defects ESR. The relation between the ESR frequencies and the magnetic field can be understood from the ground state spin Hamiltonian of the NV defect, which is written as,

$$H = h[DS_z^2 + E(S_x^2 - S_y^2)] + g\mu_B \mathbf{B}.\mathbf{S} \tag{6}$$

where z is the NV defect quantization axis, h is the Planck constant, D and E are the zero field splitting parameters, S_x, S_y and S_z the Pauli matrices, g=2.0 the Lande's g-factor, and μ_B the Bohr magneton. The $\mathbf{B}$ is the local magnetic field. The field along the NV axis can be considered as $|\mathbf{B_{NV}}| = |\mathbf{B}.\mathbf{u_{NV}}|$ with $\mathbf{u_{NV}}$ is NV center quantization axis. Therefore for any $\mathbf{B_{NV}}$, the ESR frequency is described as,

$$\Delta\nu = 2\gamma B_{NV}. \tag{7}$$

Where, γ=$g\mu_B/\hbar$ (28$\times 10^9$ $s^{-1}T^{-1}$ with g=2). To determine magnetic field at a point the Zeeman splitting in the optically detected ESR spectrum is measured. The optimal response of the spin-dependent PL signal to a DC magnetic field is obtained by fixing a driving MW frequency to the maximal slope of a given ESR dip. Due to the change in the proximity magnetic field from

the sample, the NV fluorescence rate $\frac{\partial I_0}{\partial B} \times \delta B \times \Delta t$ being I_0 is the PL intensity change and Δt is the measurement time along with the photon-noise $\sqrt{I_0 \Delta t}$. Thus the field sensitivity is given as,

$$\begin{aligned}
\eta &= \delta B \sqrt{\Delta t} \\
&= \frac{\sqrt{I_0}}{\partial I / \partial B} \\
&= \frac{4}{3\sqrt{3}} \frac{h}{g\mu_B} \frac{\Delta \nu}{C\sqrt{R_0}}.
\end{aligned} \tag{8}$$

Where $\Delta\nu$ is the ESR linewidth and C is the ESR contrast. From the experimental limit presented by the Qnami [10], the field sensitivity η is expected to be 2.3±0.2μT/$\sqrt{T}$. To record the measurements, the first step is to set up the tip-to-sample distance using the frequency modulation AFM mode where the Δf=15Hz ensures the distance $< 5nm$ to keep the best sensitivity. The microwave (MW) near-field antenna is brought in proximity (<50 μm) to the Quantilever.

There are two measurement modes possible in NV microscopy such as dual iso-B mode (where magnetic images exhibit two iso-magnetic-field (iso-B) contours [11]), which allows for rapid visualization of the magnetic textures, and then the full-B mode, which allows for a full quantitative analysis of the stray field. In dual iso-B mode, two microwave frequencies from the ESR spectra were selected, to suppress the artifacts from photoluminescence variation unrelated to the magnetic state. Here the NV center experiences the magnetic field $B_{NV}(x, y) = B_0 + B_{sample}(x, y)$ at resonance condition ($B_{NV} = \nu_{iso}/2\gamma$ and the MW induces information to NV spin and impact the fluorescence without information of the magnetic field from the sample. The magnetic field information is further recorded at each pixel and the exact value is extracted from the splitting $\delta\nu$, which is known as Full-B. The iso-B and full-B images are presented in several devices in Supplementary Figures 17, 19, 20.

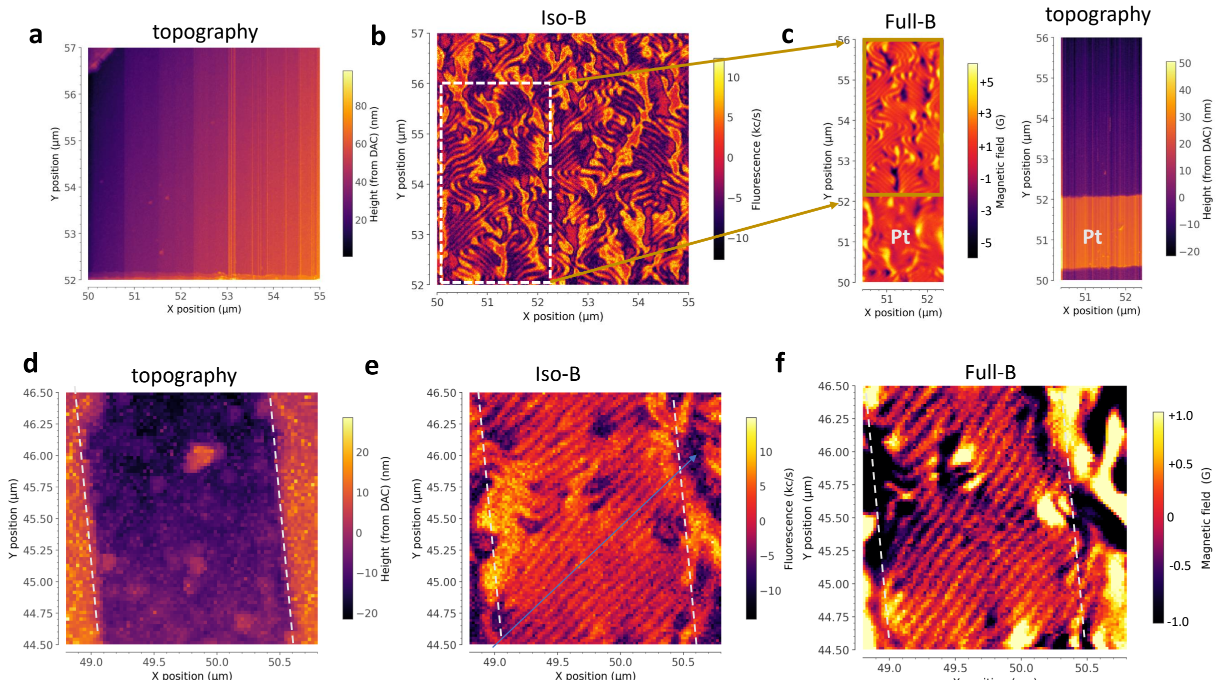

Supplementary Figure 17. **Iso-B and Full-B NV images**: **a** Topography **b** Iso-B of the film and **c** full-B around the Pt electrode to measure the stray field. **d** Topography **e** Iso-B of the poled device and **f** full-B on the poled device. The data was recorded after poling in the device [010](Figure 3d, top panel, main text). The magnetic texture is not clear behind the electrode due to screening from the Pt metal. However, the electric field line impact underneath the electrode is expected to be the same. Dotted lines indicate the edge of the Pt metal electrode on $Bi_{0.85}La_{0.15}FeO_3$ for electric field pulses.

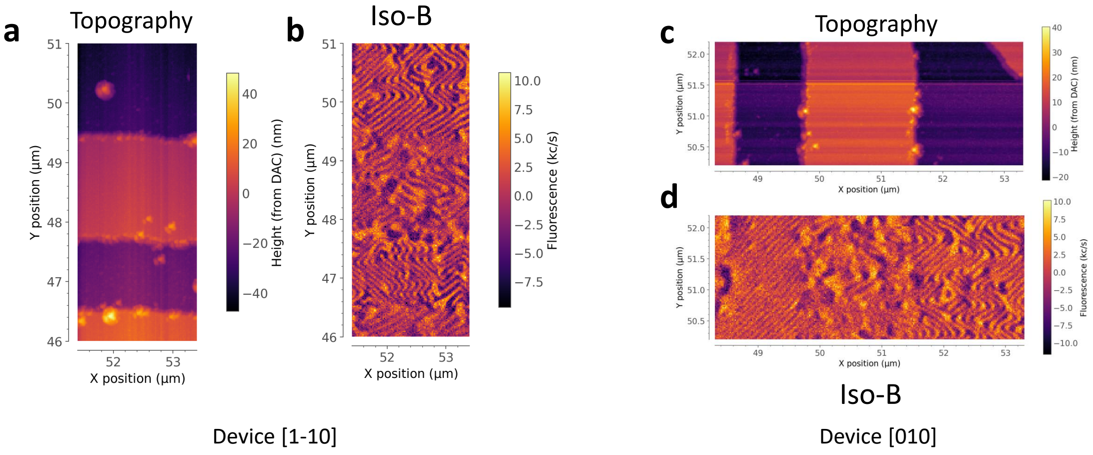

Supplementary Figure 18. **NV microscopy on different samples device for reproducibility check of the formation of the single variant in a single domain of $\mathbf{Bi_{0.85}La_{0.15}FeO_3}$ device**: **a,b** Topography of the device [100] and corresponding Iso-B NV image. **c,d** Topography of the device [010] and corresponding **f** Iso-B NV image of the magnetic texture. A single variant due to the single domain formation is consistent in all kinds of devices of $Bi_{0.85}La_{0.15}FeO_3$ thin films.

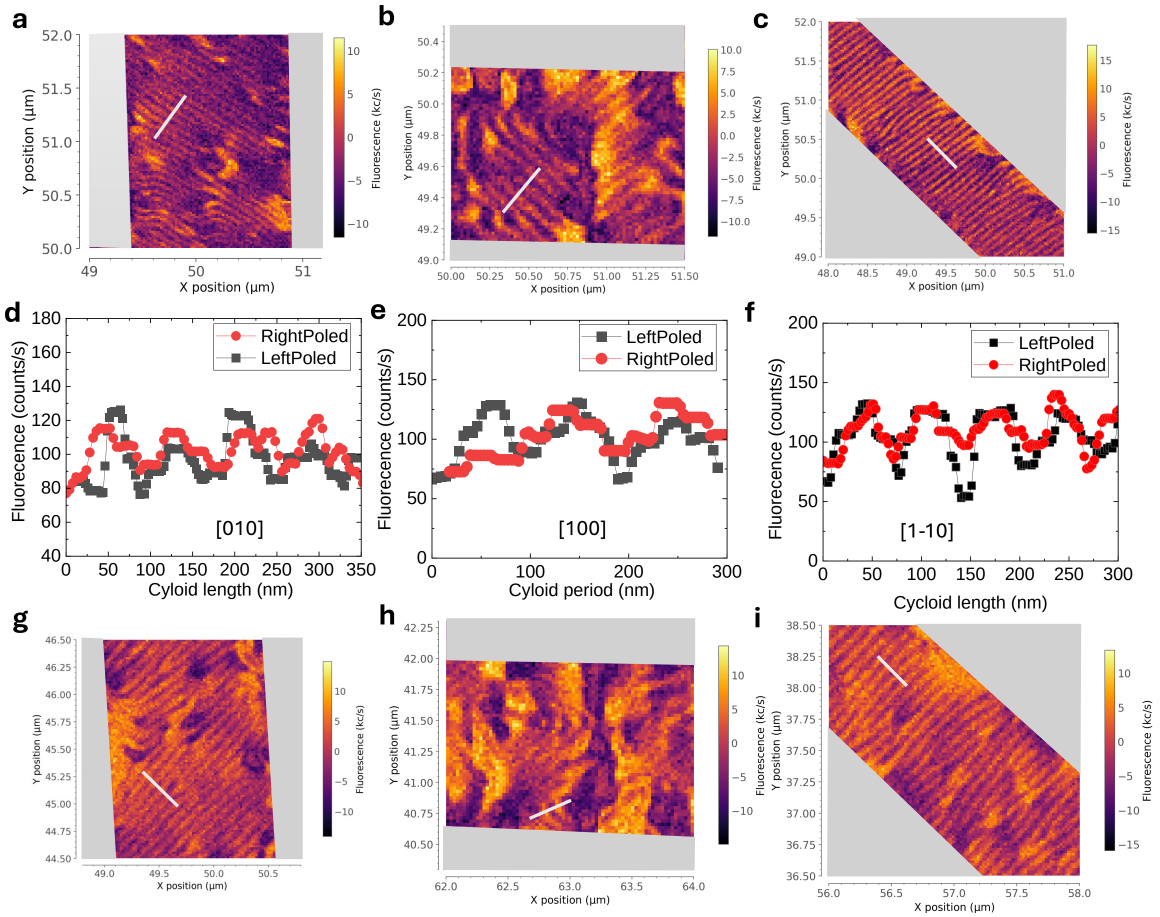

Supplementary Figure 19. Line scan for the period of the cycloid on the NV data corresponding to device **a** [010], **b** [100] and **c** [1-10] for the main text Figure 3c-d, i-j, p-q, respectively.

SUPPLEMENTARY NOTE 6

SPIN CYCLOID WAVEVECTOR AND POLARIZATION RELATION IN POLED La-BIFEO$_3$

From the NV measurement, we discovered that the presence of random ferroelectric domains in $Bi_{0.85}La_{0.15}FeO_3$ results in a combination of magnetic phases, including cycloids and the G-type antiferromagnetic phase (refer to Figure 1 in the main text and Supplementary Figure 18, 19). The former illustrates distinct variants, indicating that the magnetic states are determined by the random ferroelectric domains. The in-plane net polarization, as observed through PFM, SHG (Figure 3), is perpendicular to the cycloid propagation vector, consistent with the $BiFeO_3$

parent compound. Consequently, the cycloid is anticipated to exhibit similar characteristics.

The choice of the cycloid's propagation vector—either a single variant or two variants (type-I or type-II cycloid)—depends on the type of ferroelectric domain wall in $BiFeO_3$, as well as the boundary conditions or strain effects, as discussed in ref. [12]. On $DyScO_3$, $BiFeO_3$ prefers a bulk-like type-I cycloid with a two-variant orthogonal wave vector in 71° ferroelastic domain walls. In the case of $Bi_{0.85}La_{0.15}FeO_3$ (this work), in poled devices between the electrodes (main text Figure 3), the spin cycloid follows the single domain and represents a single variant cycloid. Supplementary Figure 17 **a**,**b** demonstrate the two possible polar states in the $\mathbf{Bi}_{0.85}\mathbf{La}_{0.15}\mathbf{FeO}_3$ unit cell along with the possible spin cycloid propagation vectors. On projecting the combination of all the vectors, the P-k orthogonal relation is only followed by the $[1-10]$ and $[110]$ based on the NV data Supplementary Figure 17 **c**,**d**. The P and k are orthogonal, therefore the $[110]$ and [1-10] are only allowed vectors in $Bi_{0.85}La_{0.15}FeO_3$ poled devices.

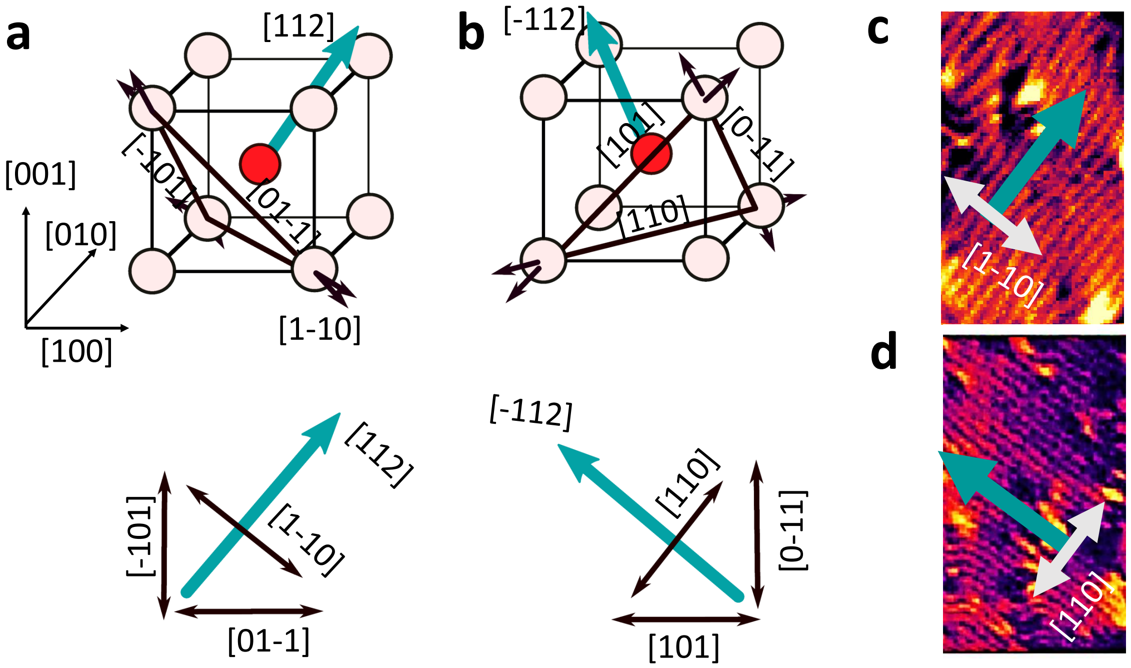

Supplementary Figure 20. **Spin cycloid propagation direction in $Bi_{0.85}La_{0.15}FeO_3$: a-b** Cycloid in two poled situations. The three possible spin cycloid propagation directions $[01\bar{1}]$ $[\bar{1}01]$ and $[1\bar{1}0]$ when the polarization is along [112]. The lower panel indicates the $[1\bar{1}0]$ is the suitable direction with relation to the polarization. When we mapped the same conditions on the NV images **c-d** the relation is testified that the P and k are orthogonal and k is along $1\bar{1}0$.

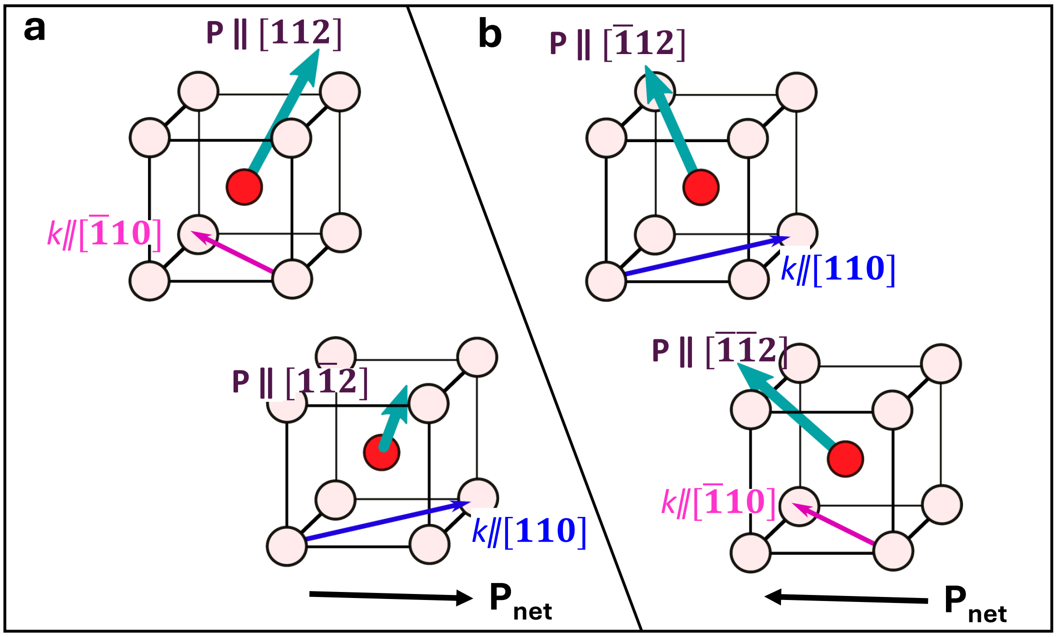

Supplementary Figure 21. Polarization and cycloid direction switching under external electric field in the device [100]. The left panel depicts the two domains with two spin cycloid propagation vectors [$\bar{1}$10] and[110] (main Fig. 2b, top panel). Right panel: after poling with electric field E, it rotates the P_{ip} from [112] to [$\bar{1}$12] (71° switch) and k switches by 90°. This is due to the strain state of the DSO substrate and therefore we do not form a single domain in the [110] direction.

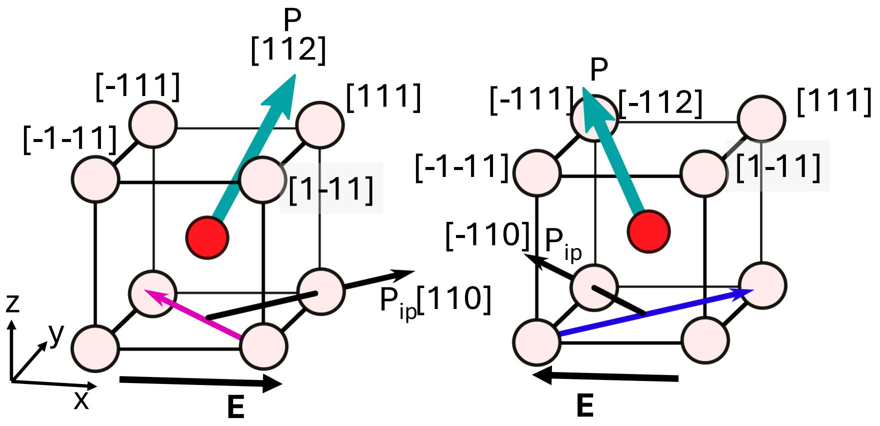

Supplementary Figure 22. Schematic indicates the direction of the polarization P, P_{ip} under in-plane electric field E.

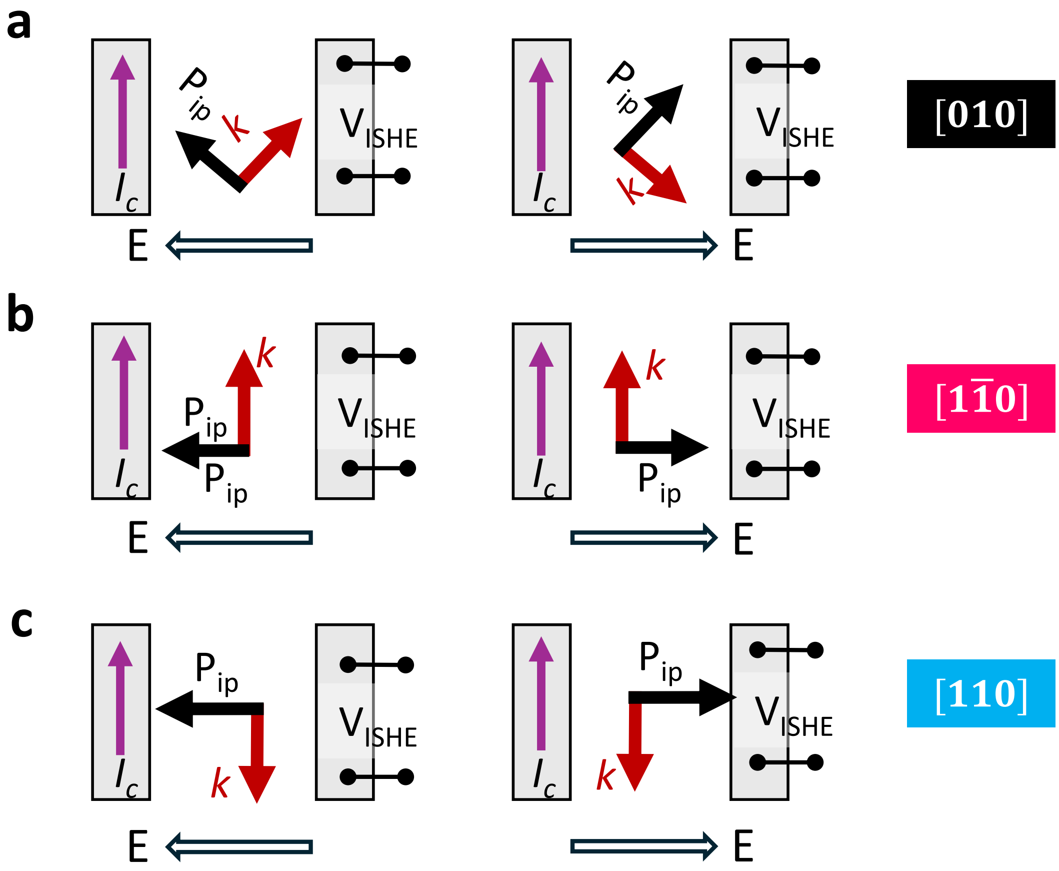

Supplementary Figure 23. **Polarization and wavevector relationship with the device orientation**: **a** [010] and **b** $1\bar{1}0$ and **c** [110]. In three cases, the Pt electrodes are rotated by $\pi/4$ with respect to the substrate edge $[001]_O$. We are not considering the case of [100] due to multidomain formation and as a result, no magnon output is detected. In single-domain orientations, the single variant cycloids are formed. Here we show that the cycloid propagation vector k has a fixed direction but due to changing the electrode orientation, the k-vector also moved with polar order as imaged in the main text Figure 3.

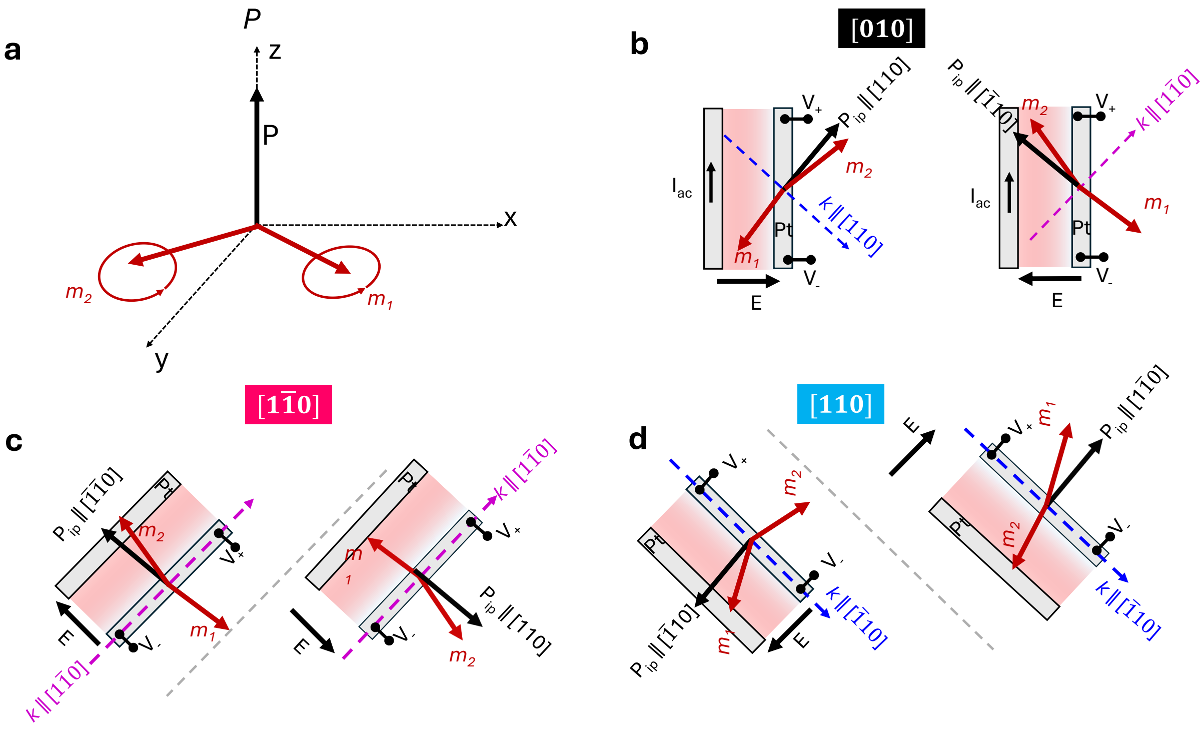

Supplementary Figure 24. **Mechanism of magnetic and polar order under electrical poling**: **a** Ferroelectric polarization with sublattice magnetization m_1, m_2. The magnetization lies in the (112) plane where the P is oriented along [112] (Figure 1, main text). The in-plane projection of P lies along $[\bar{1}10]$, [110] or $[\bar{1}\bar{1}0]$ depending on the direction of the device and P projection on the device. Schematic corresponding to the device orientation along **b** [010] and **c** $[1\bar{1}0]$ and **d** [110]. The cycloid chose the propagation direction orthogonal to P [8, 9] (Figure 3, main text), so in the case of P_{110} cycloid chose the direction $k \parallel [\bar{1}10]$. Similarly, the $P_{\bar{1}10}$ allowed $k \parallel [1\bar{1}0]$. In the polar region back and forth, the direction of P is set by the electric field and depends on the device orientation. The P is switched by $\pi/2$ in the device [010] **(b)** where the direction of k is also switched by $\pi/2$, this influences the sublattice magnetization and hence inverted upon poling, which shows the opposite ISHE voltage (Figure 4a [010]). For device $[1\bar{1}0]$, P is reversed oppositely which means the k vector remain same $[\bar{1}10]$, however as in-plane P switched by π impact m_1, m_2 as depicted in **(c)**, which shows finite hysteresis. Similarly, the hysteresis device [110] has the same but opposite effect to the device $[1\bar{1}0]$.

SUPPLEMENTARY NOTE 7

THERMALY EXCITED NON-LOCAL MAGNON TRANSPORT

In our experimental geometry, we excite the magnon in the multiferroic using a thermal gradient generated by a Pt metal wire. Our primary focus lies on thermally excited magnons to gain insights into single-domain multiferroic physics and their influence on magnon transport. Specifically, we consider the second harmonic signal for analysis. The Pt wire is heated by injecting the electrical current, and the resulting heat spreads radially (see Supplementary Figure 12a). Through the temperature gradient ΔT in the insulating magnet, the difference in magnon density between the hot and cold ends drives magnon diffusion from the hot end to the cold end. This phenomenon, governed by spin excitations or magnons, is known as the spin Seebeck effect (SSE). It's noteworthy that heat does not directly interact with magnons; rather, the temperature gradient generates phonons, and magnon-phonon scattering plays a significant role, especially at finite temperatures. All measurements are conducted at room temperature, ensuring homogeneous phonon contributions in various measurement categories. The magnon-phonon scattering contributes to relaxing magnon spin accumulation, linked to the Gilbert damping constant. Intriguingly, the thermally induced magnon excitations extend beyond the immediate source, spreading through the thermal gradient and possibly reaching several microns based on the temperature magnitude. In our study, we emphasize a relative comparison to comprehend magnon propagation in single-domain multiferroic materials. On the detector side, Pt is not considered a mere heat sink. As a consequence of the thermal gradient, a magnon chemical potential gradient forms (depletion at the injector and accumulation at the detector), initiating the transfer of magnon angular momentum beneath the detector. Due to the spin-orbit coupling (SOC) of Pt (see Supplementary Figure 12b), an open circuit condition results in the generation of

voltage due to the inverse spin Hall effect, the converse effect of the spin Hall effect is elucidated below. In the case of the second harmonic detection of the nonlocal voltage, the inverse spin Hall effect is used for electrical detection of the spin Seebeck effect. Notably, no magnetic field is employed in this setup, ruling out any contributions from the Nernst effect or anomalous Nernst effect.

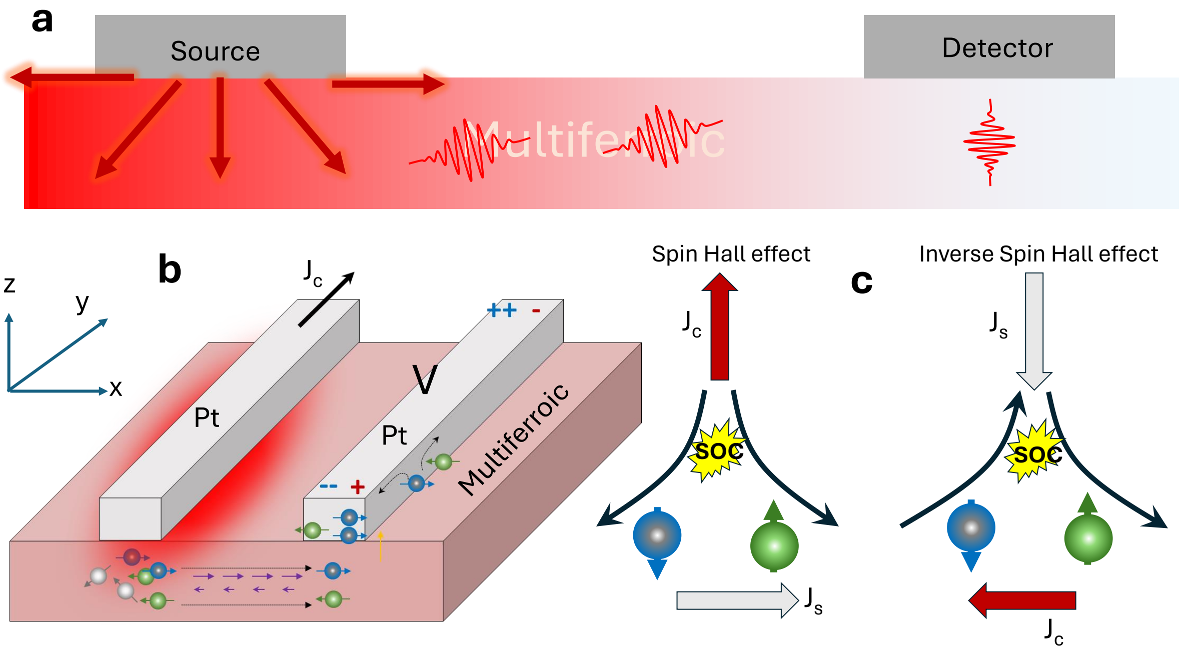

Supplementary Figure 25. **Thermal magnon transport**: **a** Nonlocal magnon transport geometry in multiferroics. **b** Spin Hall and inverse spin Hall effect.

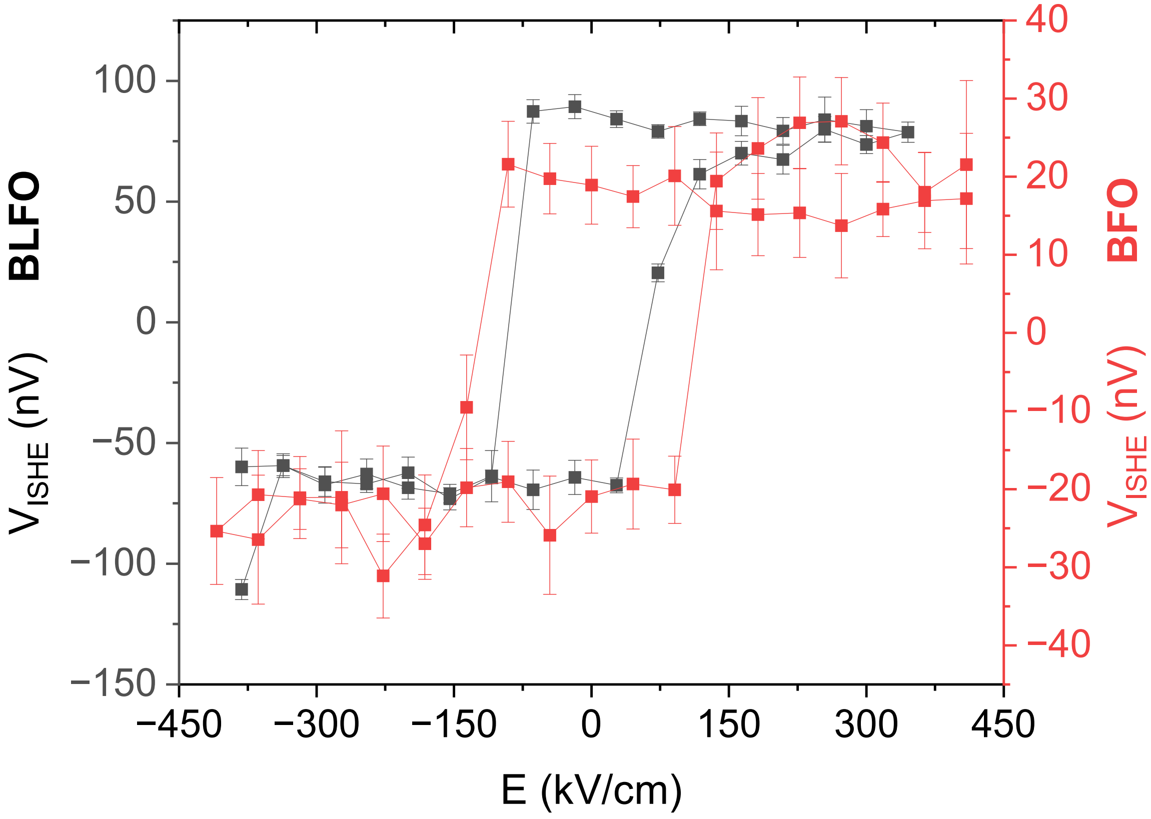

Supplementary Figure 26. Nonlocal magnon transport in $BiFeO_3$ and $Bi_{0.85}La_{0.15}FeO_3$ recorded at identical conditions. $BiFeO_3$ data is shifted to (0,0) for better comparison. $Bi_{0.85}La_{0.15}FeO_3$ data is always symmetric around zero.

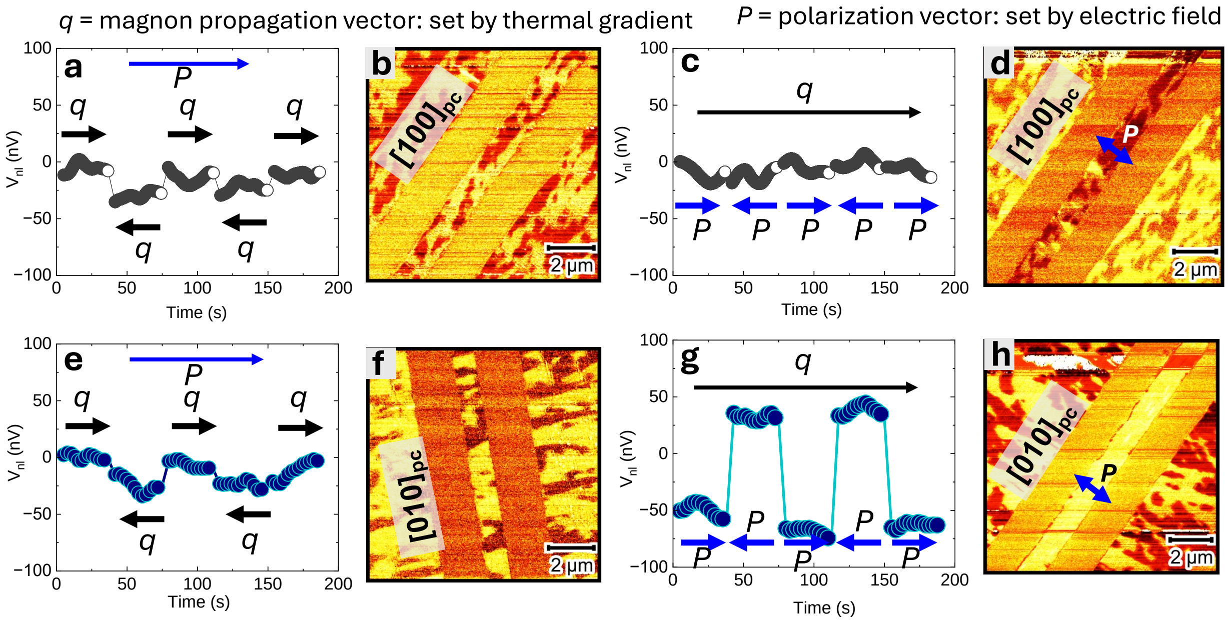

Supplementary Figure 27. **Emerging magnon transmitted voltage signal in pristine and poled $\mathbf{Bi_{0.85}La_{0.15}FeO_3}$: a-d** and **e-f** are the results on [100] and [010] devices, respectively. **a**, Nonlocal inverse spin Hall voltage (V_{ISHE}) measured in a pristine state of $Bi_{0.85}La_{0.15}FeO_3$ and corresponding PFM shown in **b**. P with a yellow arrow in **a, e** indicates the direction of polarization fixed by the ferroelectric domain (strain state of the substrate) in two orientations of devices. No external electric field was applied during the experiment in **a, b, e, f**. The experimental protocol for pristine state magnon measurement is by changing the direction of the thermal gradient (or the magnon-propagation direction) by swapping the current/voltage electrode (schematic Figure 2, main text). q represents the direction of the thermal gradient shown by the black arrow. Data in **a, e** and **c, g** is recorded under the same protocol, respectively. **c, g** is recorded under an external electric field where q remains same only the direction of P reverses. The corresponding PFM images after poling are shown in **d** and h for devices [100] and [010]. Single domain favors magnon propagation and hence the large inverse spin Hall voltage whereas multidomain does not allow magnon transmission. The power at the source electrode is fixed to 2mW corresponding to the $I_{ac} = 1.7$mA.

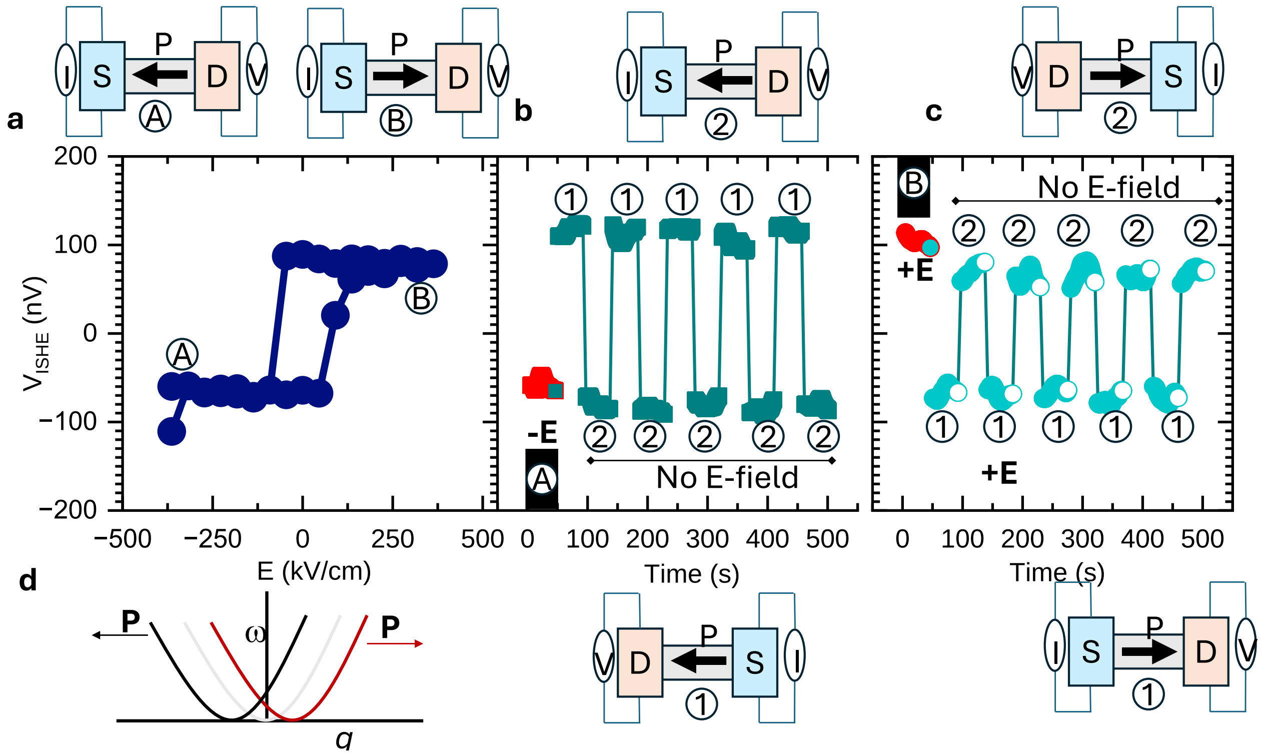

Supplementary Figure 28. **Polarization-driven non-reciprocal magnon transport: a** Magnon-electric field hysteresis, (1) and (2) represent the polarization state and source (S) and drain (D) correspond to the current source and voltage detector. **b**, the initial state is set by the electric field (in red), followed by the swapping of the electrode LEFT/RIGHT, changing the detector and source. This is done by automatically using the Keithley switch box, no physical movement of contact is involved therefore any non-reciprocity from the artifacts can be ignored. The circuit of states (1) and (2) are shown at the top and the bottom of the figure. Similarly, in the opposite polar situation in **c**, where the P direction is set by the electric field and followed by the LEFT/RIGHT S and D swapping. The difference (reversed polarity and magnitude) between the two states (**b**, **c**) is due to the non-reciprocity in $Bi_{0.85}La_{0.15}FeO_3$ imposed by the polarization state as drawn in **d**. P, polarization, ω and q represent the magnons' energy and propagation vector. Electrically, the change of the polar direction also reverses the DMI[13, 14], which imposes the non-reciprocity in the system and hence the magnon signals are different in two opposite P states, thus the polarization dependence is caused by the DMI.

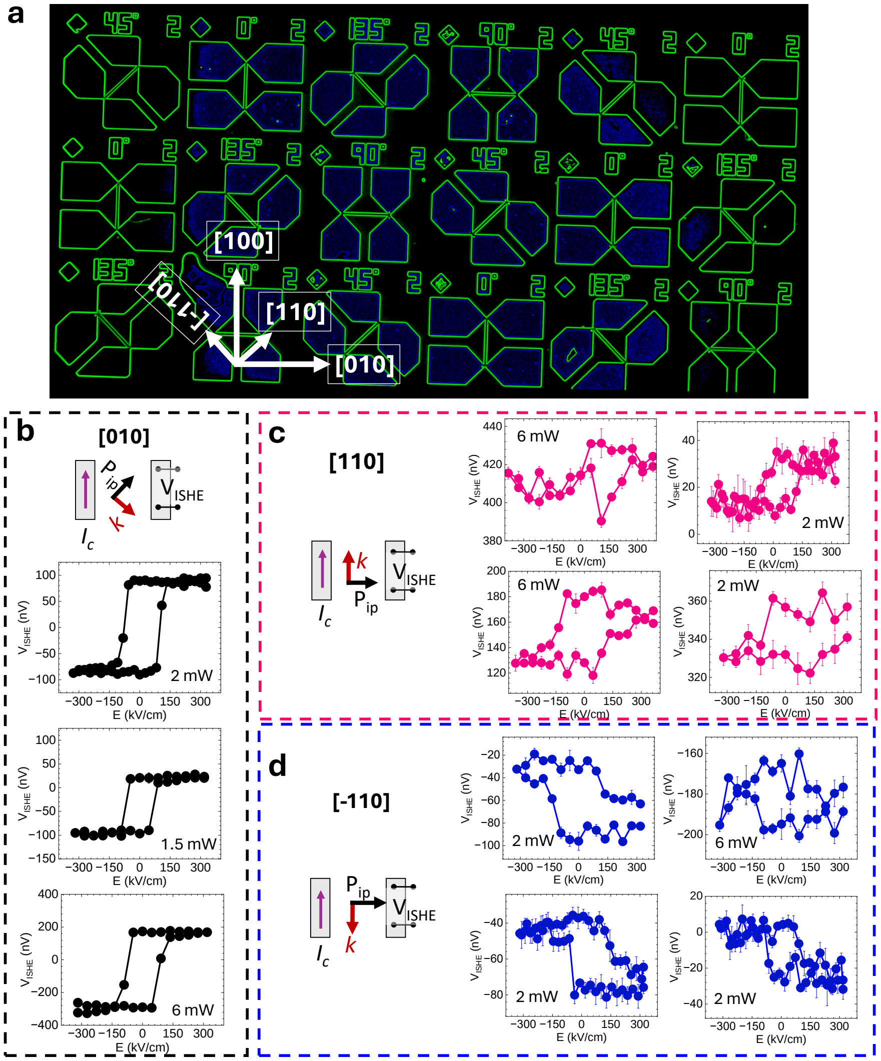

Supplementary Figure 29. **Magnon spin transport anisotropy: a** Optical image of patterned devices for spin transport measurements. 4-orientations were designed marked as 0°, 45°, 90° and 135° corresponding to pseudocubic directions [100], [110], [010], and [$\bar{1}$10], respectively. Electro-magnon hysteresis recorded in devices **b**, [010], **c**, [110] and **d**, [$\bar{1}$10]. P_{ip} and k represent the polarization and the propagation vector of the cycloid, respectively. Magnon hysteresis was recorded in different sets of devices on large-area samples. The schematics were drawn to represent the angle between the electrodes and the spin cycloid propagation vector. Irrespective of the magnitude of the nonlocal ISHE voltage (due to the different current injection) the hysteresis symmetry is preserved in three different device orientations. Device [010] is symmetric around zero whereas the ISHE voltage in [110] and [$\bar{1}$10] all the way shows positive and negative minor loops, respectively.

[1] Kavle, P. *et al.* Exchange-interaction-like behavior in ferroelectric bilayers. *Advanced Materials* **35**, 2301934 (2023).

[2] Yadav, A. *et al.* Observation of polar vortices in oxide superlattices. *Nature* **530**, 198–201 (2016).

[3] Huang, Y.-L. *et al.* Manipulating magnetoelectric energy landscape in multiferroics. *Nature communications* **11**, 2836 (2020).

[4] Boyd, R. W. Chapter 1 - the nonlinear optical susceptibility. In Boyd, R. W. (ed.) *Nonlinear Optics (Third Edition)*, 1–67 (Academic Press, Burlington, 2008), third edition edn. URL https://www.sciencedirect.com/science/article/pii/B9780123694706000010.

[5] Zhang, Y. *et al.* Ferroelectricity in a semiconducting all-inorganic halide perovskite. *Science Advances* **8**, eabj5881 (2022). URL https://www.science.org/doi/abs/10.1126/sciadv.abj5881. https://www.science.org/doi/pdf/10.1126/sciadv.abj5881.

[6] Finco, A. & Jacques, V. Single spin magnetometry and relaxometry applied to antiferromagnetic materials. *APL Materials* **11** (2023).

[7] Rondin, L. *et al.* Magnetometry with nitrogen-vacancy defects in diamond. *Reports on progress in physics* **77**, 056503 (2014).

[8] Gross, I. *et al.* Real-space imaging of non-collinear antiferromagnetic order with a single-spin magnetometer. *Nature* **549**, 252–256 (2017).

[9] Meisenheimer, P. *et al.* Switching the spin cycloid in bifeo3 with an electric field. *Nature Communications* **15**, 2903 (2024).

[10] Qnami. https://qnami.ch/. Accessed: 2024-02-07.

[11] Tetienne, J.-P. *et al.* Nitrogen-vacancy-center imaging of bubble domains in a 6-å film of cobalt

with perpendicular magnetization. *Journal of Applied Physics* **115** (2014).

[12] Haykal, A. *et al.* Antiferromagnetic textures in bifeo3 controlled by strain and electric field. *Nature communications* **11**, 1704 (2020).

[13] Rahmedov, D., Wang, D., Íñiguez, J. & Bellaiche, L. Magnetic cycloid of $BiFeO_3$ from atomistic simulations. *Phys. Rev. Lett.* **109**, 037207 (2012).

[14] Li, Z. *et al.* Multiferroic skyrmions in $BiFeO_3$. *Phys. Rev. Res.* **5**, 043109 (2023).
